# Computational insights into the stereo-selectivity of catechins for the inhibition of the cancer therapeutic target EGFR kinase

**DOI:** 10.3389/fphar.2023.1231671

**Published:** 2024-01-11

**Authors:** Mohd Rehan, Firoz Ahmed, Mohammad Imran Khan, Hifzur Rahman Ansari, Shazi Shakil, Moustafa E. El-Araby, Salman Hosawi, Mohammad Saleem

**Affiliations:** ^1^ King Fahd Medical Research Center, King Abdulaziz University, Jeddah, Saudi Arabia; ^2^ Department of Medical Laboratory Sciences, Faculty of Applied Medical Sciences, King Abdulaziz University, Jeddah, Saudi Arabia; ^3^ Department of Biological Sciences, College of Science, University of Jeddah, Jeddah, Saudi Arabia; ^4^ University of Jeddah Center for Research and Product Development, University of Jeddah, Jeddah, Saudi Arabia; ^5^ Research Center, King Faisal Specialist Hospital and Research Center, Jeddah, Saudi Arabia; ^6^ King Abdullah International Medical Research Center (KAIMRC), King Saud bin Abdulaziz University for Health Sciences, Jeddah, Saudi Arabia; ^7^ Center of Excellence in Genomic Medicine Research, King Abdulaziz University, Jeddah, Saudi Arabia; ^8^ Department of Pharmaceutical Chemistry, Faculty of Pharmacy, King Abdulaziz University, Jeddah, Saudi Arabia; ^9^ Department of Biochemistry, Faculty of Science, King Abdulaziz University, Jeddah, Saudi Arabia; ^10^ Department of Urology, Masonic Cancer Center, University of Minnesota, Minneapolis, MN, United States; ^11^ Division of Drug Metabolism and Pharmacokinetics, LabCorp Drug Development Inc., Madison, WI, United States

**Keywords:** EGCG, gallocatechin, gallocatechin gallate, small molecules, kinases, spatial isomers, diastereomers, drug delivery

## Abstract

The epidermal growth factor receptor (EGFR) plays a crucial role in regulating cellular growth and survival, and its dysregulation is implicated in various cancers, making it a prime target for cancer therapy. Natural compounds known as catechins have garnered attention as promising anticancer agents. These compounds exert their anticancer effects through diverse mechanisms, primarily by inhibiting receptor tyrosine kinases (RTKs), a protein family that includes the notable member EGFR. Catechins, characterized by two chiral centers and stereoisomerism, demonstrate variations in chemical and physical properties due to differences in the spatial orientation of atoms. Although previous studies have explored the membrane fluidity effects and transport across cellular membranes, the stereo-selectivity of catechins concerning EGFR kinase inhibition remains unexplored. In this study, we investigated the stereo-selectivity of catechins in inhibiting EGFR kinase, both in its wild-type and in the prevalent L858R mutant. Computational analyses indicated that all stereoisomers, including the extensively studied catechin (−)-EGCG, effectively bound within the ATP-binding site, potentially inhibiting EGFR kinase activity. Notably, gallated catechins emerged as superior EGFR inhibitors to their non-gallated counterparts, revealing intriguing binding trends. The top four stereoisomers exhibiting high dock scores and binding energies with wild-type EGFR comprise (−)-CG (−)-GCG (+)-CG, and (−)-EGCG. To assess dynamic behavior and stability, molecular dynamics simulations over 100 ns were conducted for the top-ranked catechin (−)-CG and the widely investigated catechin (−)-EGCG with EGFR kinase. This study enhances our understanding of how the stereoisomeric nature of a drug influences inhibitory potential, providing insights that could guide the selection of specific stereoisomers for improved efficacy inexisting drugs.

## 1 Introduction

Epidermal growth factor receptors (EGFRs) are a type of cell surface receptor belonging to the ErbB family of receptor tyrosine kinases (RTKs). EGFR is also known as ErbB/Her1 and it is the prototype of a family that also contains ErbB2/HER2/Neu, ErbB3/HER3, and ErbB4/HER4 (Pines et al., 2010). EGFRs are the receptors found anchored in the cytoplasmic membrane and are composed of an extracellular ligand-binding domain, a hydrophobic transmembrane region, and a cytoplasmic tyrosine kinase domain. EGFR signaling contributes greatly to epithelial cell growth, proliferation, and differentiation (Holgate, 2000; Schneider et al., 2009; Frey and Brent Polk, 2014). EGFRs are activated by the binding of specific ligands, such as epidermal growth factor (EGF) and transforming growth factor-α (TGF-α). The binding of the growth factor ligands to the extracellular domain of the EGFR leads to the formation of homodimers or heterodimers with other members of this family. Subsequently, the EGFR is activated through the autophosphorylation of key tyrosine residues in its cytoplasmic domain. The activated receptor provides docking sites for proteins with Src homology 2 (SH2) and phosphotyrosine binding (PTB) domains to trigger various downstream signaling pathways, including the Ras-Raf-MAPK, JAK-STAT, and PI3K-Akt pathways (Grandis and Sok, 2004; Seshacharyulu et al., 2012). This mechanism is needed for the maintenance of the epithelial cells in healthy tissue and even in inflammatory airway tissues. However, the dysregulation in EGFR signaling has been linked to various cancerous conditions (Sigismund et al., 2018). The overexpression of EGFR was observed to be a causative agent of cancer progression first identified in squamous cell carcinomas (SCCs) (Cowley et al., 1986; Olofsson et al., 1986) and then in sarcomas (Gusterson et al., 1984), non-small cell lung carcinoma (NSCLC) (Veale et al., 1987), and malignant gliomas (Wong et al., 1987). NSCLC constitutes the major part (approximately 75%) of lung cancer and the overexpression of EGFR in this cancer has been associated with drug resistance and a decreased survival rate (Ogawa et al., 1993; Veale et al., 1993; Fontanini et al., 1998; Scagliotti et al., 2004). Later, EGFR expression level was selected as a marker for predicting tumor grade, cancer progression, and early relapse of cancer (Richard et al., 1987; Salomon et al., 1987). Several studies have shown significant benefits of anti-EGFR agents in various types of solid tumors, such as head and neck cancer, NSCLC, and colorectal and pancreatic cancers, in terms of overall survival, progression-free survival, and overall response rate (Giaccone et al., 2004; Bareschino et al., 2007; Rocha-Lima et al., 2007; Petrelli and Barni, 2011; Petrelli et al., 2011). Additionally, mutations have been observed in the kinase domain of EGFR and the most common point mutation is L858R, which contributes to almost 45% of mutations in the tyrosine kinase domain (Paez et al., 2004; Shigematsu et al., 2005). The L858R mutation leads to almost 50-fold more kinase activity and higher KM for ATP than that of the wild-type EGFR (Carey et al., 2006; Yun et al., 2007). The occurrence and clinical relevance of the EGFR L858R mutation exhibit variability across distinct cancer types and diverse populations. Notably, the prevalence of the EGFR L858R mutation is prominent in NSCLC, constituting a substantial proportion of EGFR mutations within this specific cancer category (Pao et al., 2004; Hong et al., 2019). Beyond NSCLC, instances of EGFR mutations, including L858R, have been detected in select cases of head and neck squamous cell carcinoma (Stransky et al., 2011). The emergence of L858R as a potential biomarker holds promise for predicting the responsiveness of lung cancer models to anti-EGFR therapy (Marrocco et al., 2023). Notably, the use of epidermal growth factor receptor tyrosine kinase inhibitors (EGFR-TKIs) has demonstrated considerable efficacy in NSCLC patients harboring this mutation, offering a favorable side effect profile and contributing to an improved quality of life (Mitani et al., 2020).

Natural compounds hold great promise for cancer treatment and prevention. A class of natural compounds called catechins, classified within the flavonoid family and specifically categorized as flavan-3-ols or flavanols, represents a diverse group of compounds widely distributed in various fruits and vegetables. Their presence extends across a spectrum of foods, encompassing beans, apricots, strawberries, cherries, blackberries, peaches, pears, grapes, apples, black tea, green tea, red wine, cider, leafy greens, root vegetables, and legumes such as broad beans and green beans (Manach et al., 2004; Gadkari and Balaraman, 2015; Boyer and Liu, 2004; Arts et al.). Despite their ubiquity, research endeavors predominantly focus on catechins derived from green tea when examining both the concentration and inherent properties of these compounds. It is imperative to acknowledge the expansive range of dietary sources of catechins beyond green tea, as their multifaceted presence across diverse food categories underscores the potential implications for human health and nutrition. The major catechins obtained from natural sources include stereoisomers of catechin (C), gallocatechin (GC), catechin gallate (CG), and gallocatechin gallate (GCG). The anticancer activities of dietary catechins specifically from green tea have been studied in various animal models (Crespy and Williamson, 2004; Yang et al., 2011; Yang et al., 2014). To understand the molecular mechanism of anticancer activity, numerous studies with different cancer cell lines have also been carried out. The catechins act as inhibitors of receptor tyrosine kinases and exert anticancer effects by targeting the EGFR signaling pathway (Liang et al., 1997; Shimizu et al., 2005; Larsen et al., 2010; Van Aller et al., 2011; Ma et al., 2014). Most of these studies were performed on the major green tea catechin (−)-epigallocatechin-3-gallate (EGCG). The bioavailability of catechins in humans and rats was assessed following their consumption. In human studies, it was observed that EGCG ingestion led to an equal concentration of free EGCG and its key metabolite EGCG-4″-sulfate in human plasma (Hayashi et al., 2022). Another investigation in humans detected the presence of the sulfate metabolite of (−)-epicatechin along with free (−)-epicatechin in the serum (Vaidyanathan and Walle, 2002). Additionally, one study revealed that the primary metabolites found in both plasma and urine were the conjugates of (−)-epicatechin and 3′-O-methyl-(−)-epicatechin (Okushio et al., 1999). These findings collectively demonstrate that catechins, along with their key metabolites, are present in appreciable concentrations in the bloodstream after consumption. The use of the liver S9 fraction has proven to be a valuable tool for studying the metabolism of green tea catechins. Various studies have explored the bioavailability of catechins, their metabolic byproducts, and their impact on cytochrome P450 enzymes in the liver. Notably, one study indicated that non-gallated catechins do not inhibit cytochrome P450 enzymes, whereas gallated catechins exhibit inhibitory effects on all studied cytochrome P450 enzymes except CYP2D6. Among them (−)-catechin-3-O-gallate (CG) exhibited the most potent inhibition of CYP2C9 (7.60 µM), and EGCG displayed the strongest inhibition of CYP1A2 (8.93 µM) (Misaka et al., 2013; Satoh et al., 2016). Additionally, Albassam and others summarized a collection of *in vitro*, animal, and clinical studies that investigated the effect of green tea extract and its associated catechins on drug-metabolizing enzymes and drug transporters (Albassam and Markowitz, 2017). These studies collectively advance our understanding of the intricate interplay between catechins and physiological processes, highlighting their potential therapeutic relevance.

A multitude of *in vivo* investigations have provided substantial evidence supporting the cancer-preventive properties of green tea catechins. These studies have focused on diverse cancer models, encompassing breast cancer (Zan et al., 2019), pancreatic cancer (Suhail et al., 2023a), prostate cancer (Adhami et al., 2009; Khan et al., 2009), lung cancer (Chung, 1999), head and neck cancer (Kim et al.), and many more. Furthermore, the examination of catechins has extended to their potential in combination therapy with other drugs to enhance efficacy, revealing significant enhancements when drugs were employed in conjunction with catechins (Lecumberri et al., 2013; Yiannakopoulou, 2014; Vue et al., 2015). These findings collectively underscore the promising role of catechins in cancer prevention and the potential for their use in combination strategies for improved therapeutic outcomes. Computational methods, including molecular docking and binding simulation analysis, have been extensively used to gain structural insights into drug binding and to design novel drug candidates (Jamal et al., 2014; Rehan, 2015; Rehan, 2017; Rehan, 2019; Rehan et al., 2020; Rehan et al., 2021; AlZahrani et al., 2022; AlZahrani et al., 2023; Suhail et al., 2023a; Suhail et al., 2023b).

Stereoisomerism is an important phenomenon in the field of drug discovery. For a drug compound, one stereoisomer is beneficial, whereas the other stereoisomer may be harmful or lethal. The best example is that of the drug thalidomide for morning sickness in pregnant women. Thalidomide was initially prescribed as a racemic mixture (50:50 mixture of stereoisomers) without stereoisomerism being taken care of. However, it turned out that one stereoisomer of thalidomide was effective in controlling morning sickness in pregnant woman, whereas the other stereoisomer caused birth defects in the child (Dig and gle, 2001). This led to an understanding of how critical is to deal with a stereoisomeric compound. Catechins also show stereoisomerism and few studies are available on the stereo-selectivity of catechin. In one study, it was found that (−)-catechin significantly activated PPARγ in a dose-dependent fashion, whereas (+)-catechin, the stereoisomer of (−)-catechin, was not effective at all (Shin et al., 2009). Another study reported that the (+)- and (−)- stereoisomers of catechin have opposing effects on the accumulation of triglyceride induced by insulin. Using the same concentration (+)-catechin stimulated the process, whereas the (−)-catechin showed an inhibitory effect (Mochizuki and Hasegawa, 2004). Another similar study reported that (+)- and (−)- stereoisomers of catechin demonstrated opposite effects on glycogen metabolism in rat hepatocytes. The (+)-catechin inhibited glycogenolysis, whereas the (−)-catechin stimulated the process (Nyfeler et al., 1983). These examples demonstrate the importance of considering stereo-selectivity for catechin or any stereoisomeric compound while performing any activity assay. Additionally, the stereo-selectivities of catechins were studied for their membrane fluidity effects and transport across cellular membranes (Tsuchiya, 2001; Ai et al., 2019). In one of our studies, we explored the potential of green tea catechins for inhibiting the nuclear factor kappa-light-chain-enhancer of activated B cells (NF-kB), which plays a role in various cancer pathologies, including pancreatic cancer, aside from its physiological functions (Suhail et al., 2023a). Furthermore, a few studies reported the molecular docking of catechin, EGC, and EGCG with mutant EGFR (T790/L858) (Singh and Bast, 2014; Bommu et al., 2019; Minnelli et al., 2020; Sakle et al., 2020). The current study was performed with the wild-type and mutant L858R EGFR. The primary focus was to check the stereo-selectivities of catechin derivatives for the inhibition of the wild-type and mutant L858R EGFR. The study revealed the binding pose, interacting residues, molecular interactions, and dynamic behaviors of stereoisomers of major natural catechins. The binding energy and dissociation constant values were predicted, and the stereoisomers were arranged in the order of their decreasing preference for EGFR kinase inhibition. Intriguingly, we observed interesting trends in their inhibitory potential.

## 2 Materials and methods

### 2.1 Data retrieval

The three-dimensional structure coordinates of the wild-type EGFR kinase domain in complex with its bound ligand (compound 41a) and the mutant L858R EGFR kinase domain in complex with its bound ligand (PD168393) were retrieved from the protein databank (PDB) with PDB IDs of 5CAV and 4LQM, respectively. The three-dimensional structure coordinates of stereoisomers of major natural catechins were retrieved from the PubChem database with PubChem CIDs as mentioned in Table 1.

**TABLE 1:** The stereoisomers of catechin derivatives and the scores for binding strength (dock score, binding energy and pK_d_) for wild-type and mutant L858R EGFR. The higher the absolute values of the scores, the better is the binding.

Stereoisomer	PubChem CID	Binding energy (kcal/mol)		pK_d_ or −log (K_d_ ^a^)		Dock score	
		Wild-type	Mutant	Wild-type	Mutant	Wild-type	Mutant
(−)-CG	6,419,835	−8.69	−8.80	6.37	6.45	−53.07	−53.45
(−)-GCG	199,472	−8.67	−8.83	6.36	6.47	−54.60	−54.38
(+)-CG	5,276,454	−8.59	−8.78	6.30	6.44	−49.13	−49.33
(−)-EGCG	65,064	−8.58	−8.17	6.29	5.99	−49.95	−46.77
(−)-ECG	107,905	−8.53	−8.74	6.25	6.41	−48.69	−47.95
(+)-GCG	5,276,890	−8.33	−9.03	6.10	6.62	−50.66	−49.77
(+)-ECG	65,056	−8.28	−8.61	6.07	6.31	−46.93	−47.51
(+)-EGCG	2,824,823	−8.22	−8.60	6.03	6.30	−47.50	−51.35
(−)-EGC	72,277	−7.89	−7.49	5.79	5.49	−36.44	−36.48
(−)-EC	72,276	−7.82	−7.44	5.73	5.45	−35.86	−34.89
(−)-C	73,160	−7.80	−7.44	5.72	5.45	−37.50	−37.56
(−)-GC	9,882,981	−7.76	−7.70	5.69	5.64	−38.83	−37.58
(+)-GC	65,084	−7.73	−7.88	5.67	5.78	−38.29	−38.22
(+)-C	9,064	−7.70	−7.88	5.64	5.78	−36.07	−36.07
(+)-EGC	10,425,234	−7.59	−7.67	5.56	5.62	−35.74	−38.27
(+)-EC	182,232	−7.56	−7.69	5.54	5.64	−35.65	−38.35
bound References inhibitors	-	−8.89	−8.83	6.52	6.47	−59.78	−40.71

^a^ K**d**, dissociation constant.

### 2.2 Molecular docking

The molecular docking of 16 stereoisomers of catechin derivatives to the ATP-binding site of EGFR was performed by Dock v.6.5 (Ewing et al., 2001). The ATP-binding site is a crucial component of the kinase domain, and it plays a key role in the catalytic activity of the receptor. Small molecules designed to bind to this site aim to inhibit the ATP binding and, consequently, the kinase activity of EGFR. This inhibition disrupts downstream signaling pathways that are essential for cell proliferation and survival. Notably, the clinical approval of EGFR inhibitors, including gefitinib and erlotinib, which specifically bind to the ATP-binding site, serves as a compelling testament to the success of targeting this region in drug development. The dock score is a grid-based scoring method that calculates the shape and electrostatic complementary between the ligand and the receptor. The shape complementarity or steric interactions are taken care of by grid mapping, which looks for steric overlaps, contacts, and energy scoring, whereas the electrostatic interactions are estimated from an electrostatic potential (ESP) map. The ESP map is computed using the finite difference Poisson–Boltzmann equation (PBE), as used in the program DelPhi (http://compbio.clemson.edu/sapp/delphi_webserver/). In addition, the dock score also considers ligand desolvation. The ligand desolvation calculates the extent to which a ligand is buried by the binding site. Thus, the dock score calculates the best fit between the ligand and binding site using shape and electrostatic complementary and ligand desolvation, whereas the binding energy quantifies the thermodynamic strength of the interaction between a ligand and a receptor, encompassing contributions from various forces and representing the net energy change upon complex formation.

In this study, we have opted for a rigid docking option whereby the different conformations of the ligand and protein are treated as rigid objects. However, the flexibility of the ligand is taken into account by ligand conformation sampling, in which various possible conformations of the ligand are generated. For ligand docking, the binding pose of a specific conformation of the ligand with the highest absolute value of dock score is finally selected. The protein and ligand preparation required for docking were performed using Chimera v.1.6.2 (Pettersen et al., 2004). Structure visualization of protein-ligand complexes and the preparation of illustrations were performed using Pymol v.2.3.0 (The PyMOL Molecular Graphics System, 2023). In each case, the bound reference ligand was used as a probe for the active site, and the active site was identified as amino acids within the 10 Å range of the bound reference ligand.

### 2.3 Self-docking assessment

To ensure the reliability of our molecular docking and validate the results for both wild-type and mutant EGFR, we conducted self-docking of the bound reference ligands with their respective proteins (compound 41a with wild-type EGFR and mutant EGFR with PD168393). The measurement of the deviation between the docked pose and the original bound pose of the reference ligand was performed using the root-mean-square deviation (RMSD) via Pymol v. 2.3.0 (The PyMOL Molecular Graphics System, 2023).

### 2.4 Protein-ligand complex analysis

Protein-ligand interactions were analyzed and illustrations were generated using Ligplot+ *v*.1.4.5 (Laskowski and Swindells, 2011). Ligplot+ identifies hydrogen bonds and non-bonded contacts between the ligand and interacting residues of the protein. The criteria it uses for hydrogen bonds is either a distance between donor (D) and acceptor (A) atoms of ≤3.35 Å (max. D-A distance is 3.35 Å) or a distance between hydrogen (H) and acceptor (A) atoms of ≤2.70 Å (max. H-A distance is 2.70 Å), whereas the non-bonded contact is the contact between the hydrophobic atom (C or S) and any atoms that are neither covalently bonded nor interacting via hydrogen bonds and lie within the range of 2.9–3.9 Å.

### 2.5 Comparison of binding poses

We verified whether the docked catechin compounds occupied the same ligand-binding site as the bound reference ligand by comparing the binding poses of the catechin compounds with those of the bound reference ligands. Furthermore, we compared the interacting residues that were common to both the catechin compounds and the bound reference ligands. Additionally, we predicted the binding energy and dissociation constant terms for the ligand-protein complexes using the independent software Xscore v. 1.2.11 (Wang et al., 2003).

### 2.6 *In silico* alanine scanning mutagenesis

*In silico* alanine scanning mutagenesis for a given protein-ligand complex is performed to determine the contribution of all binding site residues in ligand binding. Each binding site residue is mutated to alanine and then the binding energy is calculated for each mutant. All these calculations were performed using the Alanine Binding Site-Scan (ABS-Scan) tool (Anand et al., 2014). The program by default considers the binding site as all the residues within the cutoff of 4.5 Å of the ligand, and these residues are selected for mutation. Finally, by comparing their binding energies with the wild-type, we may know the contribution of each residue in binding. In this study, we selected those residues that showed a binding energy loss of ≥0.3 kcal/mol upon mutation.

### 2.7 Molecular dynamic simulation

A molecular dynamic (MD) simulation was carried out using Gromacs v.2019.6 (Abraham et al., 2015) with the charmm36-feb2021 force field. The topology file of the ligand was generated by the CGenFF server (https://cgenff.umaryland.edu/) using the charmm force field. The proteins were solvated in a dodecahedron unit cell at an edge distance of 1 nm from the surface of the protein. The water used for solvation was a simple point charge water molecule, spc216. The solvated system was neutralized by adding counter ions, and energy minimization was performed using the steepest descent method. To avoid surface effects, periodic boundary conditions were used. After energy minimization, the system was subjected to NVT (constant number of particles, volume, and temperature) and then NPT (constant number of particles, pressure, and temperature) equilibrations at 300 K and 1.0 bar for 100 ps each. The time step of each simulation was set to 2 fs. The equilibrated systems were finally run for 100 ns (500, 00, 000 steps), and the trajectories were saved every 10 ps (5,000 steps).

The MD simulation trajectory was analyzed by using various methods to check the stability of the protein-ligand complexes, as detailed in the Results section. One such analysis was hydrogen bond occupancy analysis, which was performed to assess the stability of hydrogen bonds. This is the fraction of conformations in which a particular hydrogen bond is formed throughout the MD simulation run time. This analysis was performed using the “gmx hbond” command of Gromacs followed by the use of the Python script “readHBmap.py”, which is available at GitHub (https://github.com/quytruong1808/vilas/blob/master/vilas/analyzer/readHBmap.py).

## 3 Results

### 3.1 Library of stereoisomers of catechin derivatives

**FIGURE 1:**
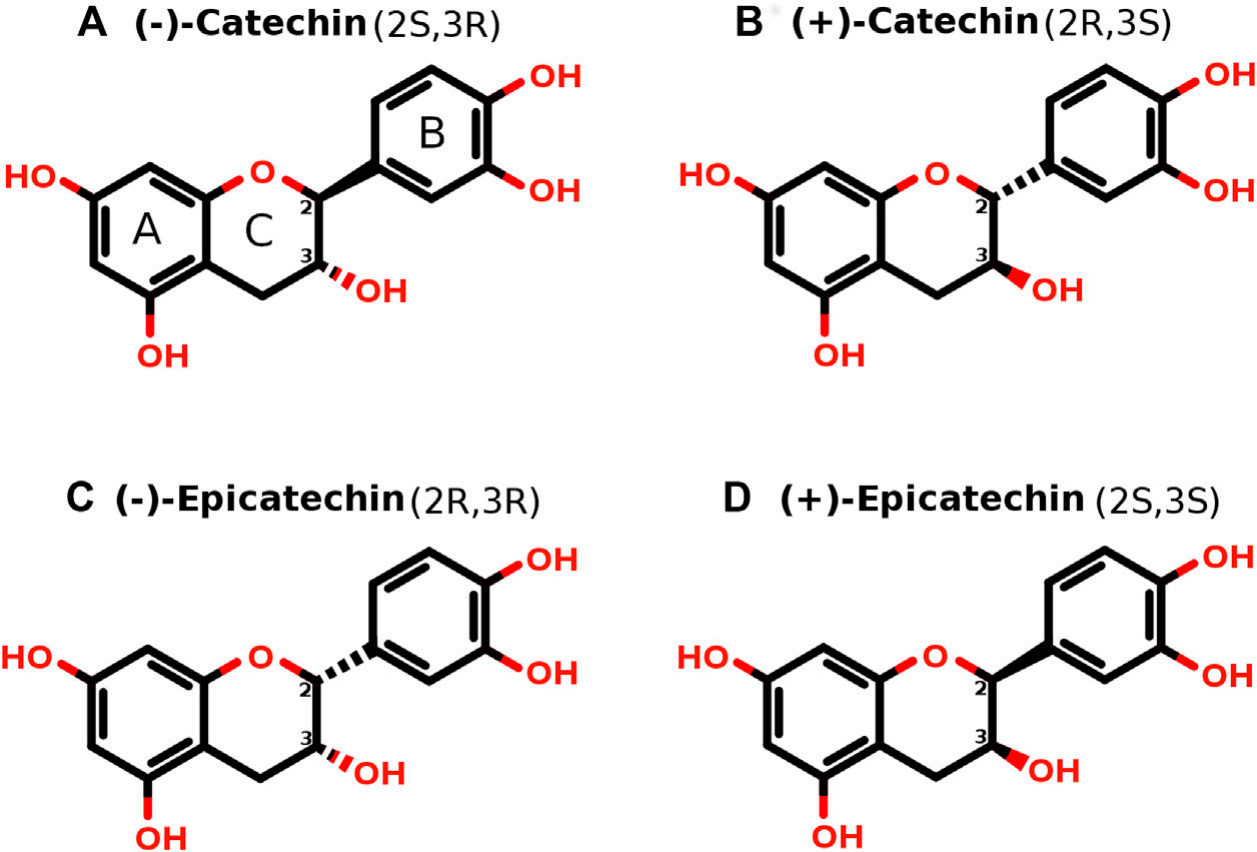
**(A–D)** Four stereoisomers of catechin. Two chiral centers are shown on carbon 2 and carbon 3. The stereoisomers vary in the positioning of chemical groups at carbon 2 and carbon 3. The solid bond indicates that it is protruding toward us, whereas the dashed bond indicates that it is going away from us. The “R” (Latin: “rectus”, means right) and “S” (Latin: “sinister”, means left) notations for the “2” and “3” positions of the chiral carbons are shown for each stereoisomer. Based on these two bonds at carbon 2 and carbon 3, there are two trans isomers called catechin, shown in panel **(A)** and panel **(B)**, and two cis isomers called epicatechin, shown in panel **(C)** and panel **(D)**.

**FIGURE 2:**
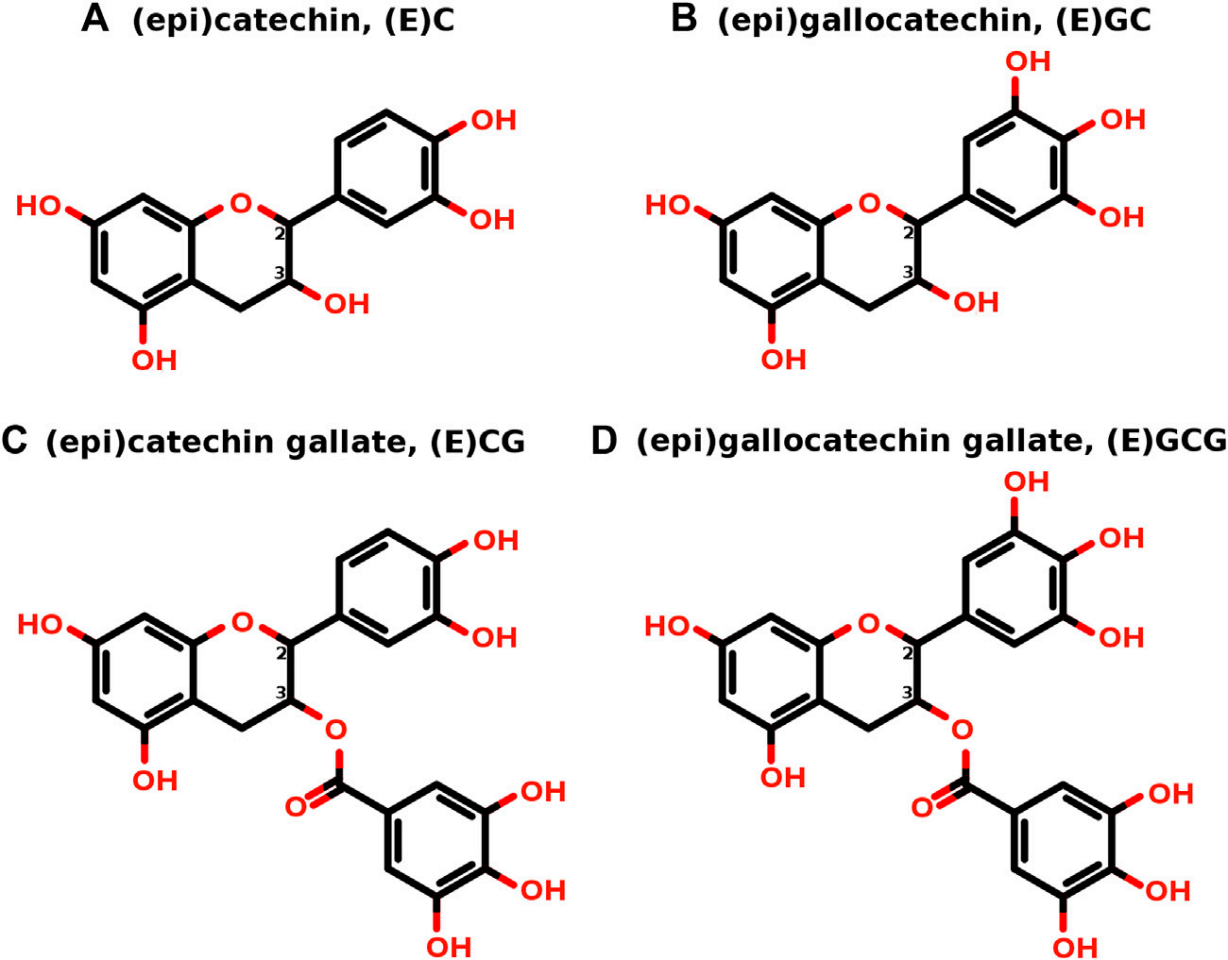
**(A–D)** The two-dimensional structures of the four major natural catechins. The chiral centers are shown as carbon 2 and carbon 3. Based on the orientation of the bonds joining other chemical groups to these chiral carbons, every catechin derivative has two trans isomers and two cis (epi) isomers, making a total of four stereoisomers.

Catechin possesses two benzene rings, rings A and B, and a heterocyclic ring, ring C (Figure 1A), and contains two chiral centers on carbon 2 and carbon 3 and forms two trans isomers (catechin) and two cis isomers (epicatechin), resulting in a total of four stereoisomers (Figure 1). There are four major natural catechin derivatives (Figure 2) and each catechin derivative can have four stereoisomers, thus making the total count 16. Having the same molecular formula and chemical groups, cis and trans isomers generally have the same chemical properties; however, the different orientations of chemical groups often lead to different physical properties and biological activities. The structures of all 16 stereoisomers of catechin derivatives were retrieved from PubChem and further explored for EGFR kinase inhibition.

### 3.2 Self-docking assessment

**FIGURE 3:**
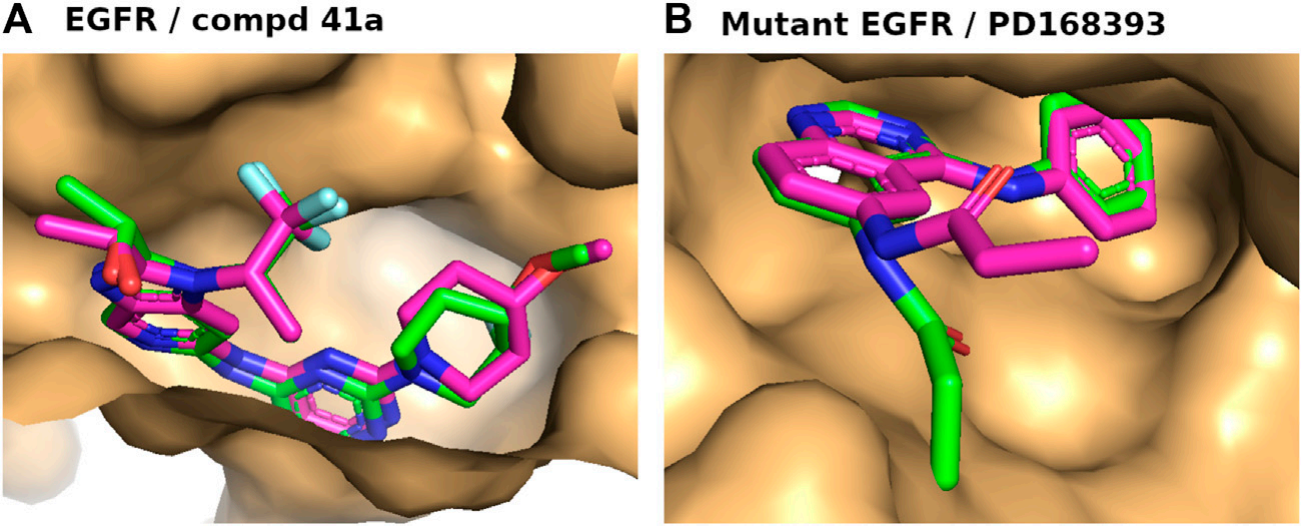
**(A, B)** Self-docking analyses of the bound reference ligands, compound 41a for wild-type EGFR **(A)** and PD168393 for mutant EGFR **(B)**. The ligand binding sites of the proteins are depicted as surfaces in light orange, with the docked pose of the bound reference ligand with backbone in pink and the original bound pose in green.

We conducted a self-docking analysis to validate the quality of molecular docking for the selected three-dimensional structures and ensure the accuracy of our docking results. In this evaluation, we determined the RMSD value for all matching atoms between the docked pose and the original pose of the bound reference ligand with the target protein. For the wild-type, the docked pose closely matched the original pose, resulting in an RMSD value of 0.71 (Figure 3A). Similarly, for mutant EGFR, the docked pose and the original pose exhibited a close alignment, with an RMSD value of 0.73 (Figure 3B). Notably, there was a slight change in the conformation of the bound reference ligand’s tail during docking, leading to a slight increase in the RMSD value, although it remained within the accepted range of 2 Å RMSD for high-quality docking. This confirmed the reliability of our docking procedure and indicated that the three-dimensional structures of these proteins were well-suited for molecular docking and exploring the binding poses of other ligands.

### 3.3 Molecular docking analyses of all 16 stereoisomers of catechins with wild-type EGFR

**FIGURE 4:**
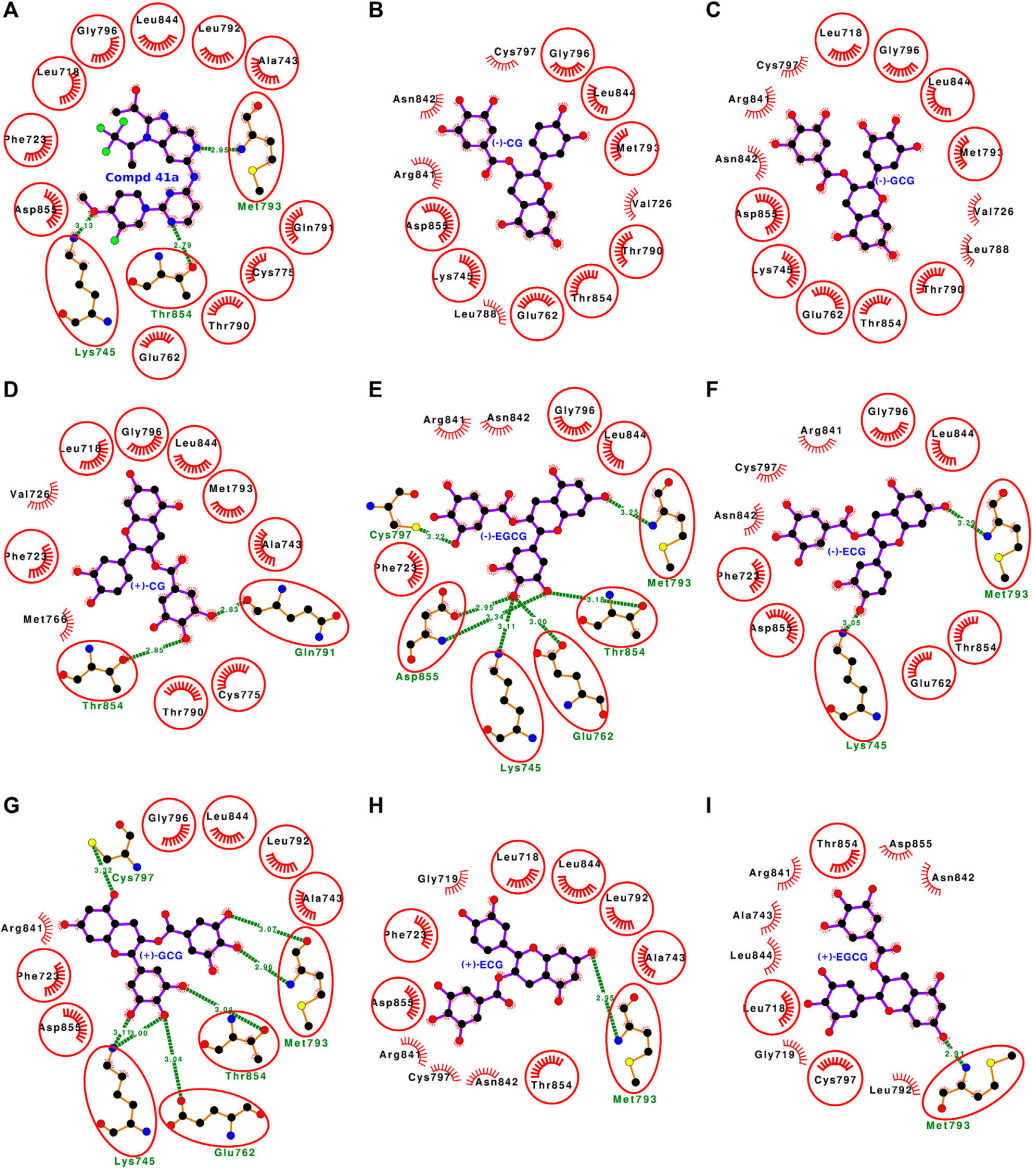
**(A–I)** Protein-ligand interaction plots of the bound reference ligand (compound 41a) and the top eight ranked stereoisomers of catechin derivatives with wild-type EGFR. The amino acid residues forming hydrophobic interactions are shown as comb-like structures with bristles. The interacting residues in common with those of the bound reference ligand are encircled. The ligand and the residues forming hydrogen bonding interactions are shown as ball-and-stick representations. The color of the balls distinguishes various atom types: the black balls represent carbon atoms, the red balls represent oxygen atoms, the blue balls represent nitrogen atoms, the yellow balls represent sulfur atoms, and the green balls represent fluorine atoms. The hydrogen bonds are shown as green dashed lines labeled with bond lengths (in Å).

**FIGURE 5:**
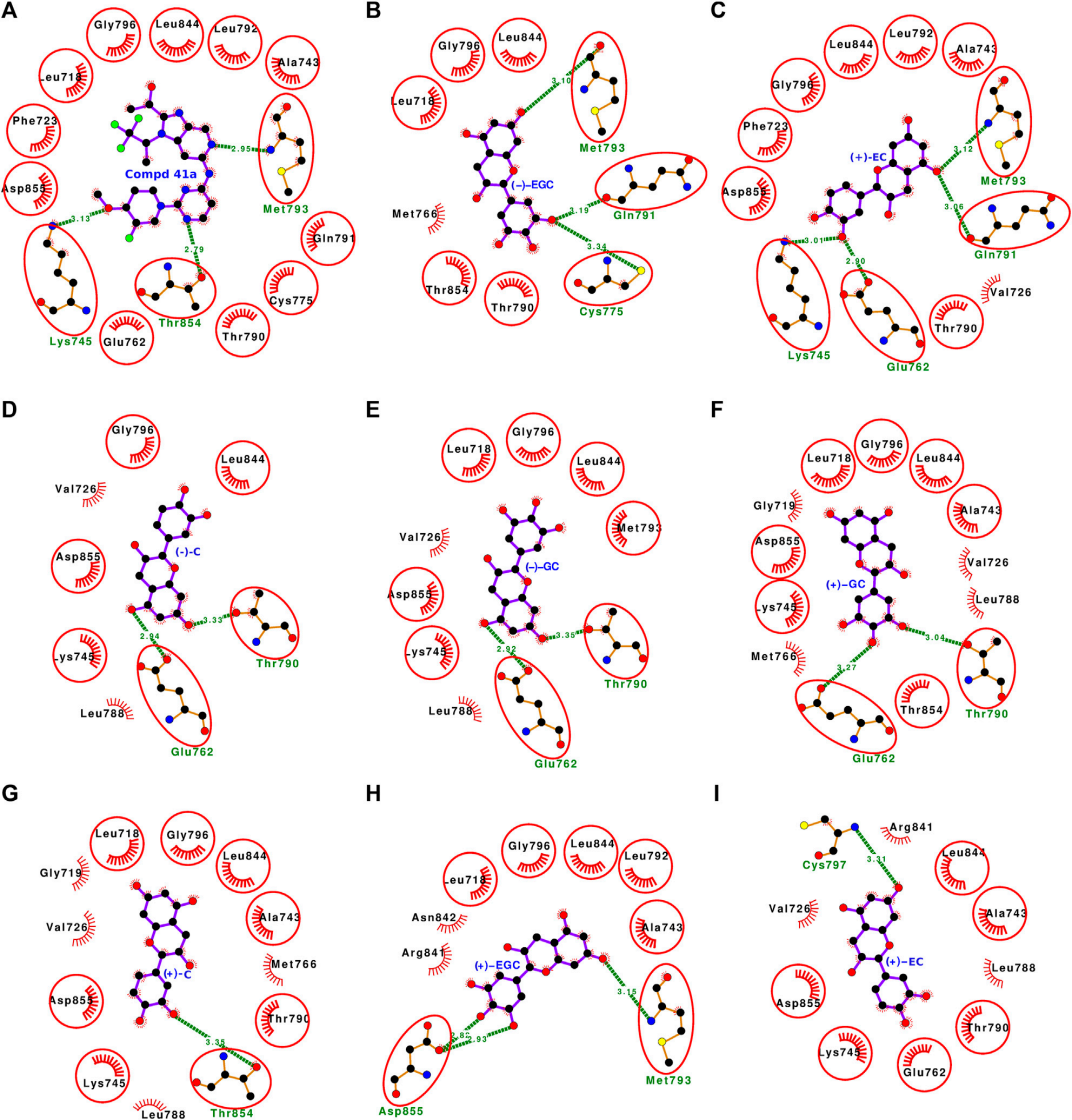
**(A–I)** Protein-ligand interaction plots of the bound reference ligand (compound 41a) and the bottom eight ranked stereoisomers of catechin derivatives with wild-type EGFR. The amino acid residues forming hydrophobic interactions are shown as comb-like structures with bristles. The interacting residues in common with those of the bound reference ligand are encircled. The ligand and the residues forming hydrogen bonding interactions are shown as ball-and-stick representations. The color of the balls distinguishes various atom types: the black balls represent carbon atoms, the red balls represent oxygen atoms, the blue balls represent nitrogen atoms, the yellow balls represent sulfur atoms, and the green balls represent fluorine atoms. The hydrogen bonds are shown as green dashed lines labeled with bond lengths (in Å).

**FIGURE 6:**
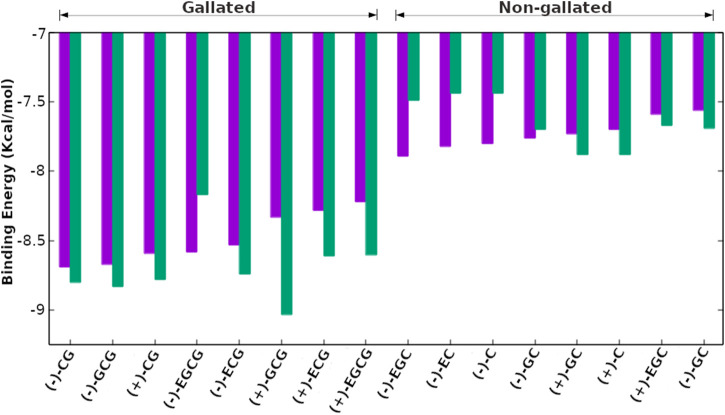
The stereoisomers of catechin derivatives with their binding energy scores for wild-type (purple) and mutant L858R (green) EGFR. The binding energies of stereoisomers for the wild-type shown as purple bars are arranged in decreasing absolute values of binding energies. The gallated catechin derivatives clustered together and stood out with the non-gallated ones in having higher scores for the binding energies for both the wild-type and the mutant EGFR.

The molecular docking of all 16 stereoisomers of four catechins with wild-type EGFR revealed that all of them bound to the binding site and might inhibit EGFR kinase activity (Supplementary Figure S1, Figures 4, 5). The dock scores for the stereoisomers varied from −35.65 to −54.60 (Table 1). However, the binding preference of these stereoisomers varied based on the binding energy, which varied from −7.56 to −8.69 kcal/mol. All the stereoisomers were arranged in the order of decreasing binding energy (Figure 6; Table 1). For the dissociation constant, pK_d_ (−logK_d_) values were presented ranging from 5.54 to 6.37. The stereoisomers were found interacting with 8–14 residues of EGFR, and all the interacting residues for each stereoisomer are listed in Supplementary Table S1. The number of interacting residues found in common with the interactive residues of the bound reference ligand varied from 6 to 11. The number of hydrogen bonds formed by these stereoisomers varied from 1 to 7 except for the first two ranked compounds (−)-CG and (−)-GCG, which showed no hydrogen bonding (Figures 4, 5).

### 3.4 Molecular docking analyses of all 16 stereoisomers of catechins with mutant L858R EGFR

**FIGURE 7:**
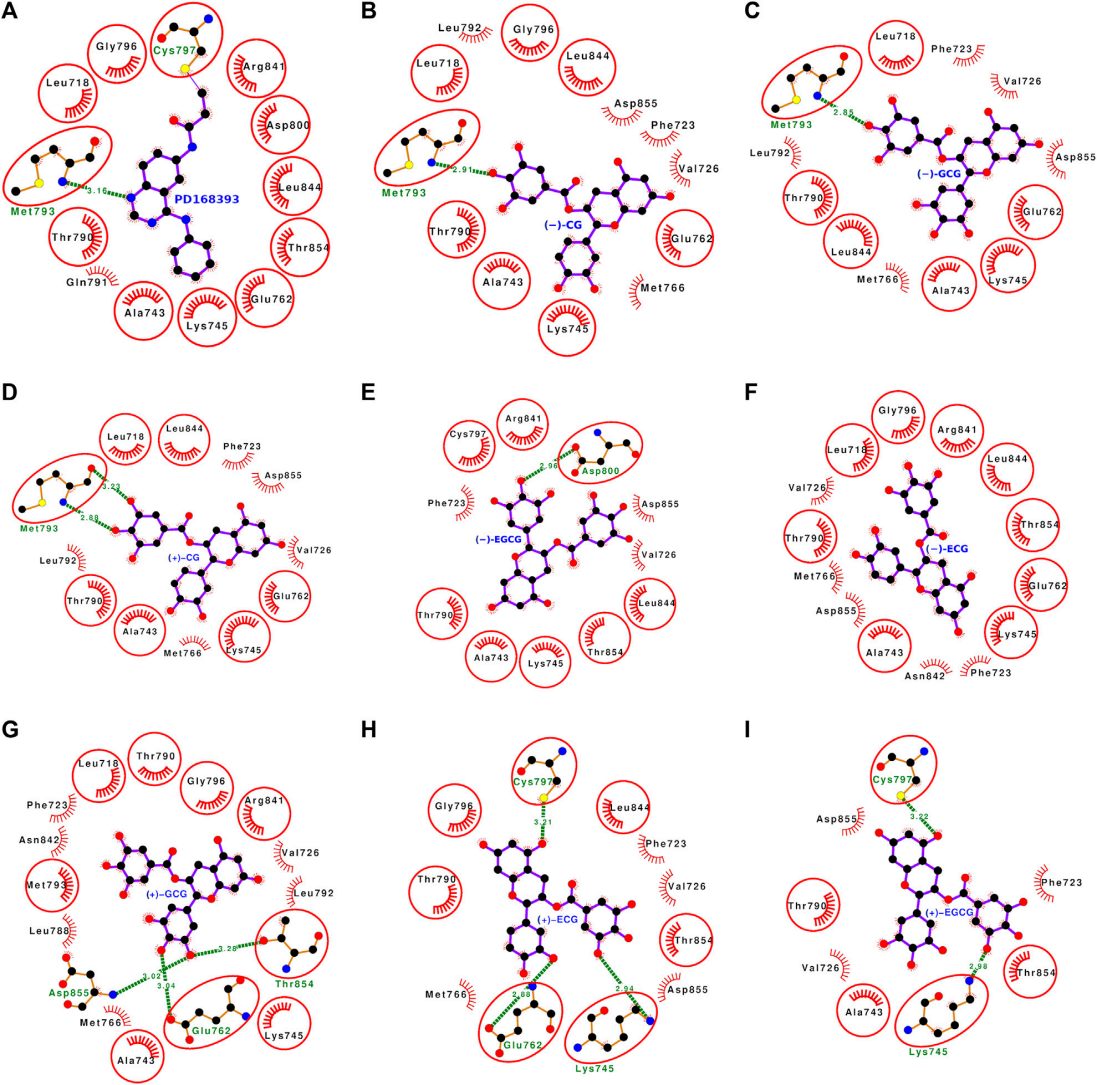
**(A–I)** Protein-ligand interaction plots of the bound reference ligand (PD168393) and the stereoisomers of catechin derivatives with mutant L858R EGFR. The order of stereoisomers appearing in this figure is the same as in Figure 4. The amino acid residues forming hydrophobic interactions are shown as comb-like structure with bristles. The interacting residues in common with those of the bound reference ligand are encircled. The ligand and the residues forming hydrogen bonding interactions are shown as ball-and-stick representations. The color of the balls distinguishes various atom types: the black balls represent carbon atoms, the red balls represent oxygen atoms, the blue balls represent nitrogen atoms, the yellow balls represent sulfur atoms, and the green balls represent bromine atoms. The hydrogen bonds are shown as green dashed lines labeled with bond lengths (in Å).

**FIGURE 8:**
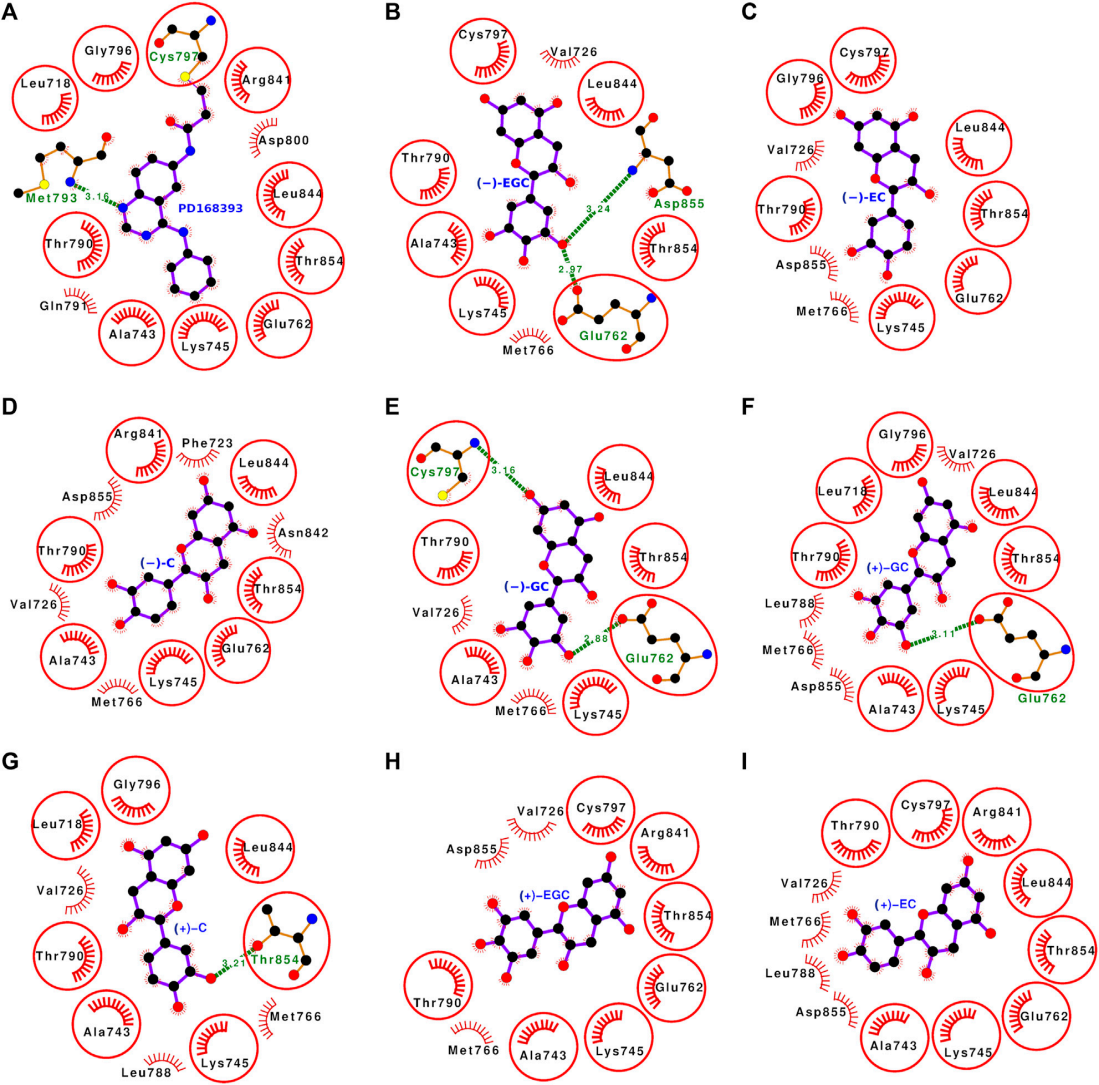
**(A–I)** Protein-ligand interaction plots of the bound reference ligand (PD168393) and the stereoisomers of catechin derivatives with mutant L858R EGFR. The order of stereoisomers appearing in this figure is the same as in Figure 5. The amino acid residues forming hydrophobic interactions are shown as comb-like structure with bristles. The interacting residues in common with those of the bound reference ligand are encircled. The ligand and the residues forming hydrogen bonding interactions are shown as ball-and-stick representations. The color of the balls distinguishes various atom types: the black balls represent carbon atoms, the red balls represent oxygen atoms, the blue balls represent nitrogen atoms, the yellow balls represent sulfur atoms, and the green balls represent bromine atoms. The hydrogen bonds are shown as green dashed lines labeled with bond lengths (in Å).

The molecular docking with mutant L858R EGFR also showed that all the 16 stereoisomers bound well within the binding site (Figures 7, 8). The dock score ranged from −34.89 to −54.38 and the binding energy was within the range of −7.44 to −9.03 kcal/mol. The dissociation constant term pK_d_ (−logK_d_) value was falling between 5.45 and 6.62 (Table 1). The stereoisomers were interacting with the EGFR L858R mutant through 8–16 interacting residues, and all the interacting residues for each stereoisomer are listed in Supplementary Table S2. The number of interacting residues found in common with the interacting residues of the bound reference inhibitor ranged from 5 to 9. The hydrogen bonds formed by the stereoisomers were within 0–3 bonds. All gallated stereoiosmers formed hydrogen bonds except for (−)-ECG (Figure 7).

### 3.5 Molecular docking analyses of the first rank catechin derivative stereoisomer for wild-type (−)-CG

The first rank catechin derivative stereoisomer for wild-type EGFR was (−)-CG. The docking results showed that it bound well within the ATP-binding site (Figure 4B, Supplementary Figure S2A). The binding strength scores, including the dock score (−53.07), the binding energy (−8.69 kcal/mol), and the dissociation constant related term (pK_d_: 6.37), were also high (Table 1). The (−)-CG in the ATP-binding site interacted with the following 13 residues: Asp-855, Thr-854, Leu-844, Asn-842, Arg-841, Cys-797, Gly-796, Met-793, Thr-790, Leu-788, Glu-762, Lys-745, and Val-726 (Figure 4B). These 13 interacting residues formed 37 non-bonded contacts and thus stabilized the complex (Table 2). Among the interacting residues, Arg-841 played a key role in binding as it was involved in the maximum number of interactions (11 non-bonded contacts). When the binding of (−)-CG was compared with that of the bound reference inhibitor, eight residues were shared within the list of interacting residues of both ligands. This suggests that (−)-CG also blocks the same set of residues and inhibits kinase activity like the bound reference inhibitor.

**TABLE 2:** Molecular interactions of (−)-CG binding to wild-type EGFR kinase. All the interacting residues are listed with the number of non-bonded contacts.

Interacting residue	Non-bonded contacts
Val-726	2
Lys-745	6
Glu-762	2
Leu-788	2
Thr-790	3
Met-793	1
Gly-796	1
Cys-797	1
Arg-841	11
Asn-842	3
Leu-844	1
Thr-854	1
Asp-855	3

### 3.6 Molecular docking analyses of (−)-CG with mutant L858R EGFR

(−)-CG docked well with mutant L858 EGFR and showed high binding strength scores, including the dock score (−53.45), binding energy (−8.80 kcal/mol), and dissociation constant term pK_d_: 6.45 (Table 1). All of these scores for (−)-CG with the mutant EGFR were higher than the respective scores with wild-type EGFR. (−)-CG bound well within the mutant binding site and interacted with the following 13 residues: Leu-718, Phe-723, Val-726, Ala-743, Lys-745, Glu-762, Met-766, Thr-790, Leu-792, Met-793, Gly-796, Leu-844, and Asp-855 (Figure 7B, Supplementary Figure S2B). These 13 interacting residues of the mutant EGFR formed 42 non-bonded contacts and a hydrogen bond (Table 3). Among the interacting residues, Lys-745 and Met-793 played a key role in binding. Lys-745 formed the maximum number of non-bonded contacts (10) and Met-793 formed a hydrogen bond and three non-bonded contacts. The comparison of (−)-CG binding with the binding of the bound reference inhibitor showed that eight interacting residues were shared among the binding poses of both ligands. This suggests that (−)-CG also inhibits mutant L858R EGFR like the bound reference inhibitor.

**TABLE 3:** Molecular interactions of (−)-CG binding to mutant L858R EGFR. All the interacting residues are listed with the number of hydrogen bonds and non-bonded contacts.

Interacting residue	Hydrogen bonds	Non-bonded contacts
Leu-718		1
Phe-723		4
Val-726		3
Ala-743		1
Lys-745		10
Glu-762		1
Met-766		4
Thr-790		2
Leu-792		1
Met-793	1	3
Gly-796		1
Leu-844		5
Asp-855		6

### 3.7 Comparative binding pose analyses of (−)-CG with wild-type and mutant EGFR

**FIGURE 9:**
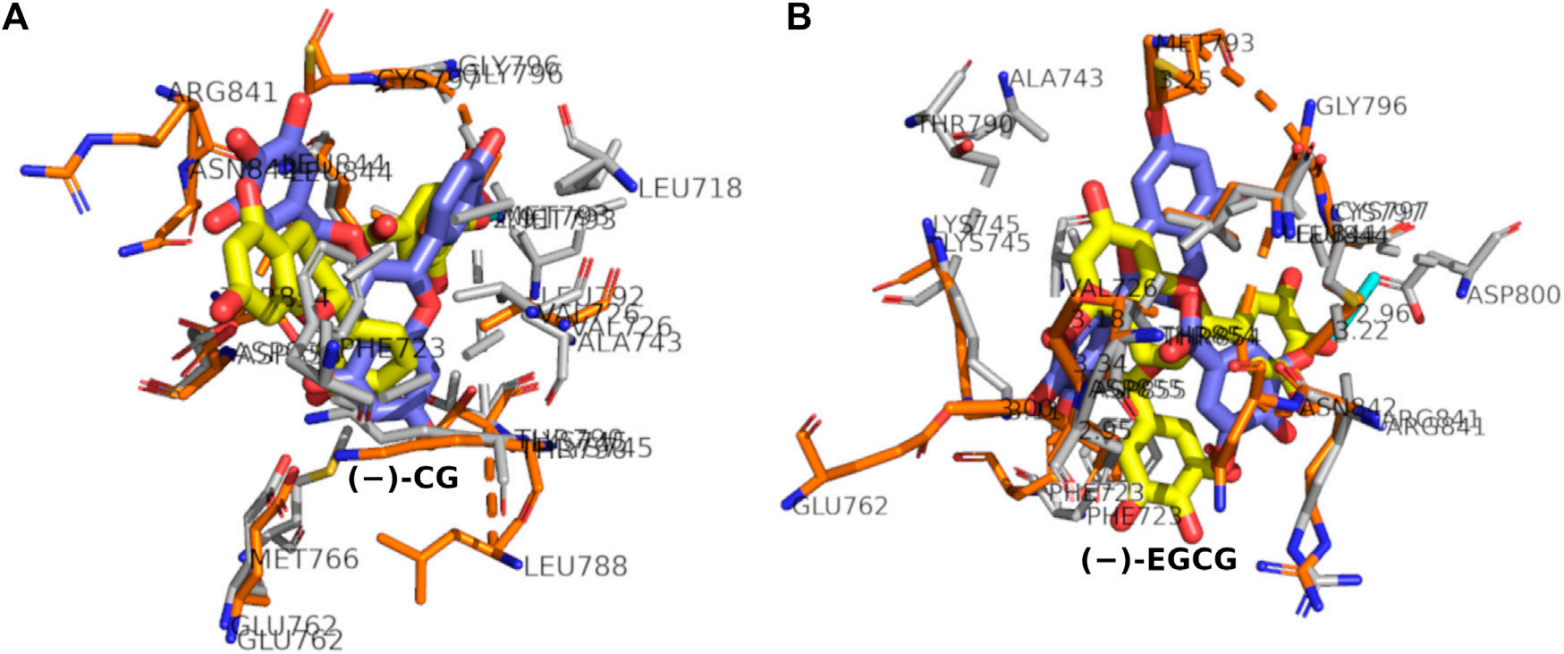
**(A,B)** Superposition of binding poses of (−)-CG **(A)** and (−)-EGCG **(B)** withwild-type and mutant EGFR. The ligands (labeled with the ligand name) and interacting residues (gray, labeled with the residue name) are shown in stick representations. The ligand bound with wild-type EGFR is shown in blue, while the one bound with mutant EGFR is shown in yellow. The heteroatoms of the ligands and interacting residues are shown in standard colors (e.g., O-atom, red; N-atom, blue). Hydrogen bonds are shown in cyan and labeled with bond length (in Å).

The comparative analysis of binding poses of (−)-CG in wild-type and mutant protein showed that (−)-CG bound to the same site and interacted with 13 residues with eight residues in common and five varying residues (Figure 9A). The binding strength scores of (−)-CG with wild-type and mutant protein were comparable. In the case of the mutant, there were 42 non-bonded contacts (with five added) and a hydrogen bond (newly added). In addition, a slight change in the orientation of (−)-CG was observed. Overall, the (−)-CG was binding to the same site involving a similar set of residues with comparable binding strength scores. This showed that there was not much variation in the binding affinity of (−)-CG upon mutation.

### 3.8 Molecular docking analyses of the most studied and major tea catechin (−)-EGCG with wild-type EGFR

(−)-EGCG is one of the most studied catechins and is the major constituent of green tea. It ranked four of the 16 stereoisomers and the binding energy was comparable with the first rank compound in wild-type EGFR docking (Table 1). The docking study showed that the compound bound well within the ATP-binding site and interacted with the following 11 amino acid residues: Phe-723, Lys-745, Glu-762, Met-793, Gly-796, Cys-797, Arg-841, Asn-842, Leu-844, Thr-854, and Asp-855 (Figure 4E, Supplementary Figure S2C). These 11 interacting residues formed 28 non-bonded contacts and seven hydrogen bonds that stabilized the protein-ligand complex (Table 4). Furthermore, the absolute values of the dock score (−49.95), binding energy (−8.58 kcal/mol), and dissociation constant term pK_d_ (6.29) were also high (Table 1). Of the 11 interacting residues, six residues, Lys-745, Glu-762, Met-793, Cys-797, Thr-854, and Asp-855, were also involved in hydrogen bond formation in addition to non-bonded contacts. The residue Asp-855 played a key role in binding as it was involved in the maximum number of non-bonded contacts (7) and the maximum number of hydrogen bonds (2). When comparing (−)-EGCG binding with that of the bound reference inhibitor, the majority of the eight interacting residues (the encircled residues in Figure 4E) were also on the list of interacting residues for the bound reference inhibitor. This implies that (−)-EGCG is also blocking the same set of residues and thus inhibiting the EGFR kinase like the bound reference inhibitor.

**TABLE 4:** Molecular interactions of (−)-EGCG binding to wild-type EGFR. All the interacting residues are listed with the number of hydrogen bonds and non-bonded contacts.

Interacting residue	Hydrogen bonds	Non-bonded contacts
Phe-723		1
Lys-745	1	3
Glu-762	1	1
Met-793	1	3
Gly-796		2
Cys-797	1	1
Arg-841		7
Asn-842		1
Leu-844		1
Thr-854	1	1
Asp-855	2	7

### 3.9 Molecular docking analyses of (−)-EGCG with mutant EGFR

The docking analysis of (−)-EGCG with the EGFR L858R mutant showed that it bound well within the binding site and interacted with the following 11 residues: Phe-723, Val-726, Ala-743, Lys-745, Thr-790, Cys-797, Asp-800, Arg-841, Leu-844, Thr-854, and Asp-855 (Figure 7E, Supplementary Figure S2D). These 11 interacting residues formed 41 non-bonded contacts and a hydrogen bond (Table 5). Among the interacting residues, Phe-723 and Asp-800 played key roles in binding as the former formed the maximum number of non-bonded contacts (12) and the latter formed a hydrogen bond and three non-bonded contacts. A comparison of the binding pose of (−)-EGCG with that of the bound reference inhibitor showed eight common residues among the interacting residues of both ligands. The binding strength scores, dock score (−46.77), binding energy (−8.17 kcal/mol), and dissociation constant term pK_d_ (5.99) were also high, indicating protein-ligand complex stability (Table 1). However, the binding strength scores of (−)-EGCG were less than those of the binding with wild-type. This suggests that the L858R mutation in EGFR leads to a small decrease in (−)-EGCG binding.

**TABLE 5:** Molecular interactions of (−)-EGCG binding to mutant L858R EGFR. All the interacting residues are listed with the number of hydrogen bonds and non-bonded contacts.

Interacting residue	Hydrogen bonds	Non-bonded contacts
Phe-723		12
Val-726		1
Ala-743		1
Lys-745		1
Thr-790		1
Cys-797		5
Asp-800	1	3
Arg-841		4
Leu-844		7
Thr-854		2
Asp-855		4

### 3.10 Comparative binding pose analyses of (−)-EGCG with wild-type and mutant EGFR

The binding pose of (−)-EGCG with wild-type EGFR was compared with that of the mutant EGFR. It was observed that (−)-EGCG bound to the same site and interacted with same number of residues (11), with common seven residues and four varying residues in both the wild-type and mutant cases. In the case of the wild-type (−)-EGCG formed 28 non-bonded contacts and seven hydrogen bonds (Figure 9B), whereas, in the case of the mutant, it formed 41 non-bonded contacts and a hydrogen bond. Thus, a loss of six hydrogen bonds (strong interactions) with a gain of non-bonded contacts (weak interactions) occurred upon mutation. In addition, a slight change in the orientation of (−)-EGCG and a subtle decrease in binding strength scores were observed. Thus, the decrease in binding upon mutation may be attributed to a change in the active site conformation upon distal mutation.

### 3.11 *In silico* alanine scanning mutagenesis

In this study, the binding site residues that showed a binding energy loss of ≥0.3 kcal/mol upon mutation were selected and determined to contribute toward binding energy. In (−)-CG binding with wild-type EGFR, five residues were picked up as being involved in binding using *in silico* alanine scanning mutagenesis. However, all five identified residues had a binding energy loss of ≥0.4 kcal/mol upon mutation. The list included Val-726, Lys-745, Arg-841, and Leu-844 from the interacting residues. This list included the key residue Arg-841 (a maximum number of non-bonded contacts of 11). In addition, the residue Leu-718, which was not among the identified interacting residues, was picked up.

In (−)-CG binding with mutant EGFR, five residues were identified as contributing to binding energy. The list included Phe-723, Val-726, Lys-745, Leu-844, and Asp-855. All the picked residues were from the identified interacting residues. The key residue Lys-745 (a maximum number of non-bonded contacts of 10) was also picked up in the scanning.

In (−)-EGCG binding with wild-type EGFR, six residues were picked up as contributing enough toward (−)-EGCG binding. The list included four residues from the interacting residues (Phe-723, Lys-745, Arg-841, and Leu-844) and two other residues, Leu-718 and Val-726. The picked interacting residues included Lys-745, which formed a hydrogen bond and three non-bonded contacts, and Arg-841, which had the maximum number of non-bonded contacts (7). Another key residue, Asp-855, which had the maximum number of non-bonded contacts (7) and two hydrogen bonds, was not picked up here; however, it showed a 0.25 kcal/mol (cutoff, 0.3 kcal/mol) decrease in binding energy upon mutation.

In (−)-EGCG binding with the mutant EGFR, five residues, Phe-723, Val-726, Lys-745, Leu-844, and Asp-855, were picked up through alanine scanning mutagenesis analysis. All the picked residues were from the identified interacting residues. Phe-723 was the key residue as it had the maximum number of non-bonded contacts (12). Additionally, in this scan, Phe-723 was picked up with the highest binding energy loss upon mutation.

### 3.12 Molecular dynamic (MD) simulation analyses

To gain more insights into the stability and dynamics of the interactions of (−)-CG and (−)-EGCG with wild-type and mutant EGFR, MD simulations were performed. The simulation results were analyzed by comparing and calculating various measures, including RMS deviation of the protein backbone, RMSF values for each residue, hydrogen bond number, and the radii of gyration.

#### 3.12.1 RMS deviation of the protein backbone

**FIGURE 10:**
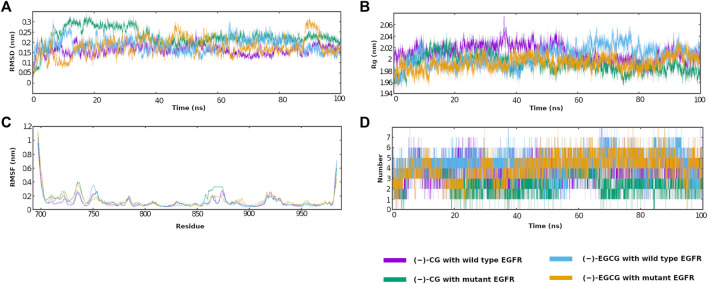
**(A–D)** MD simulation analyses for the docked complexes of (−)-CG and (−)-EGCG with wild-type and mutant EGFR. **(A)** RMS deviations (RMSD) of the protein backbone with the simulation time for the protein-ligand complexes. **(B)** Variations in radii of gyration (Rg) with the simulation time for the protein-ligand complexes. **(C)** The fluctuations of the residues with the simulation time for the protein-ligand complexes. **(D)** Number of hydrogen bonds between the ligands and the protein with simulation time.

To check the stability of the protein-ligand complex, a root-mean-square deviation (RMSD) of the backbone atoms of the protein was calculated for various time intervals during the simulation run of 100 ns. For complexes of (−)-CG with wild-type and mutant EGFR, the RMSD curves were comparable, and both showed an initial increase in RMSD, followed by a plateau. However, the (−)-CG complex with the mutant had a slightly higher RMSD than the wild-type during MD simulation, and the average RMSDs of the protein backbone for the entire simulation run for (−)-CG complex with wild-type and mutant protein were 0.21 and 0.28 nm, respectively (Figure 10A), whereas the RMSD curves of the (−)-EGCG complex with wild-type and mutant EGFR were similar and both showed an initial increase, and then a plateau was almost reached. The average RMSDs of the protein backbone of (−)-EGCG with wild-type and mutant protein for the total simulation run time were 0.24 nm and 0.23 nm, respectively (Figure 10A). The RMS deviations of all the four complexes (two with wild-type and two with mutant) were well within the acceptable limits (0.2–0.3 nm). This suggested that all the protein complexes did not significantly deviate from the reference docked complexes and were stable in the MD simulation.

#### 3.12.2 Radii of gyration (Rg) analysis

An alternative approach to check the stability of the protein is the radii of gyration (Rg), which measures the size and compactness of the protein. The Rg values of the complexes of (−)-CG and (−)-EGCG with wild-type and mutant were comparable and did not vary much. The average Rg values for all the four complexes (two with wild type and two with mutant) were calculated to be 2.0 nm each (Figure 10B). The low variation in the Rg value means that the protein does not unfold or loosen, which again indicates the good stability of all four protein-ligand complexes.

#### 3.12.3 RMS fluctuations of amino acids

RMSF estimates the flexibility and rigidity of various parts of the protein. For a stable protein-ligand complex, the fluctuation of the residues within the binding site should be restricted. The RMS fluctuations were not high for all the amino acids of the protein in all four complexes. However, little peaks were observed for two stretches of amino acid residues, 700–755 and 864–924, for all the four complexes (Figure 10C). This lack of fluctuations for the whole protein, particularly the binding site, indicated that few conformational changes occurred after ligand binding and the protein-ligand complexes were stable.

#### 3.12.4 Hydrogen bond number

The analysis of hydrogen bonds during MD simulation is also important for determining the binding specificity and stability of the protein-ligand complex. The hydrogen bonding pairs of donors and acceptors within a 0.35 nm distance were calculated. This 100 ns run included 10,000 frames as coordinates, and the energy and log files were written for each 10 ps (=0.01 ns) constituting a frame. The hydrogen bonds per frame averages for (−)-CG with wild-type and mutant were 3.83 and 2.62, respectively, whereas the hydrogen bonds per frame averages for (−)-EGCG with wild-type and mutant were 4.54 and 4.05, respectively. It was observed that the ligand binding was stable enough due to the reasonable number of hydrogen bonds during the 100-ns MD simulation (Figure 10D). The hydrogen bonds during MD simulation were further analyzed through a hydrogen bond occupancy analysis.

#### 3.12.5 Hydrogen bond occupancy analyses

Hydrogen bond occupancy is the fraction of conformations in which a particular hydrogen bond is formed throughout the varying conformations in the total MD simulation run time. The raw file showing total hydrogen bond pairs with occupancy is provided as Supplementary Table S3. The analysis revealed that (−)-CG binding with wild-type protein showed no hydrogen bonding in the docked pose; however, a few hydrogen bonds formed with varying occupancy during the MD simulation. The residue Met-793 alone showed hydrogen bonding through different atoms, and in two cases, with 89% and 60% occupancy. The reason for this is that in the initial docked pose, the distance was slightly more than the required cutoff for the hydrogen bonding.

(−)-CG binding with mutant protein in the docked pose showed a hydrogen bond through Met-793. Hydrogen bond occupancy analysis revealed that this bond was had 57.2% occupancy throughout the 100 ns simulation, and this was the only hydrogen bond with more than 50% occupancy. This showed the stability of the hydrogen bond during the course of the 100 ns MD simulation. In addition, a few other hydrogen bonds were formed with varying occupancy.
(−)-EGCG binding with wild-type protein in the docked pose showed seven hydrogen bonds through six residues. However, the hydrogen bond occupancy results showed low occupancy or no bond for all cases except Met-793, which showed a strong hydrogen bond with high occupancy (occupancy >60%). In addition, many other hydrogen bonds formed with varying occupancy during the MD simulation. This includes three hydrogen bonds with ≥50% occupancy involving two residues, Glu-762 (two bonds with occupancies of 50% and 53%) and Thr-790 (occupancy, 51%), which stabilized the protein-ligand complex.
(−)-EGCG binding with mutant protein in the docked pose showed a hydrogen bond through Asp-800. The hydrogen bond occupancy analysis showed that this bond had the highest occupancy (approximately 50% occupancy) and thus it was stable during the MD simulation. In addition, a few other H-bonds were observed with varying occupancy.

In general, with wild-type protein, Met-793 showed hydrogen bonding for (−)-CG and (−)-EGCG with more than 50% occupancy during the MD simulation, whereas with the mutant protein, it showed hydrogen bonding with more than 50% occupancy for (−)-CG only, but no hydrogen bond formation for (−)-EGCG during the MD simulation.

## 4 Discussion

The present study investigated the stereo-selectivity of catechin derivatives for EGFR kinase inhibition using computational methods (mainly molecular docking). The self-docking analyses of the bound reference ligands for both wild-type and mutant EGFR affirmed the quality of docking and the reliability of the results. Additionally, they demonstrated the compatibility of the selected three-dimensional structures with our docking software, making them suitable for exploring the binding poses of other ligands through molecular docking. Furthermore, the analysis of the binding poses of catechin derivatives, along with their comparison with bound reference ligands, revealed consistent binding to the same site as the bound reference ligand, along with interactions involving a similar set of residues, despite the generous search space (10 Å around the bound reference ligand). Additionally, the binding energy and dissociation constant values, calculated using independent software, aligned with the dock score. Collectively, these convergent findings strongly supported the quality of our docking method. The study found that the stereoisomers docked with wild-type and mutant L858R EGFR and were arranged in decreasing order of absolute values of binding energy with respect to the wild-type case. The highest-ranking stereoisomer (−)-CG and the most studied one (−)-EGCG in complex with wild-type and mutant EGFR were analyzed using various techniques and presented in detail. The binding poses of (−)-CG and (−)-EGCG with wild-type were superposed with the respective mutant binding poses and comparative analyses for the binding location, interacting residues, and the orientations of the ligands were performed. There was not much variation except for a slight change in the orientation of the ligand. The interacting residues were also emphasized using *in silico* alanine scanning mutagenesis and the hydrogen bonds were analyzed using occupancy analysis in the MD simulation. In general, the gallated catechins (E)CG and (E)GCG showed a higher affinity toward EGFR kinase inhibition than the non-gallated catechins (E)C and (E)GC. Thus, the affinity increased with the 3-gallate esterification of non-gallated catechins. The molecular docking of the stereoisomers with mutant EGFR also showed the same pattern, i.e., the gallated catechins showing a higher affinity than the non-gallated catechins. The first rank catechin derivative stereoisomer for wild-type EGFR was (−)-CG and its binding was increased with mutant L858R EGFR. Interestingly, the binding energy of the gallated catechins increased more with mutant L858R EGFR than with wild-type EGFR, except for (−)-EGCG. To sum up, these stereoisomers were inhibiting wild-type EGFR and the mutant L858R EGFR. Residue 858, which is mutated in the mutant version, was not picked up as an interacting residue in wild-type or the mutant for any stereosiomer. This observation suggests that the mutation in the 858 residue is not directly affecting the binding of ligands but the change in binding is observed due to a change in the conformation of the binding site as a result of distal mutation. It is noteworthy that the stereoisomers, especially those that are gallated, are suggested to be inhibiting the mutant EGFR more effectively than the wild-type. Therefore, the stereoisomers are proposed to be effective in inhibiting both the wild-type and mutant L858R EGFR. The trans and cis isomers of a derivative with wild-type EGFR docking had similar binding energies. However, a study on the transport of catechin stereoisomers through the membrane of Caco-2 cells (Ai et al., 2019) shows that the trans catechins are displaying better transcellular permeability than their corresponding cis (epi) catechins and, thus, trans catechins are fluxed more into the lumen after absorption in humans. Therefore, this study of the membrane transportation suggests that cis isomers (epicatechins) may have better oral bioavailability than trans catechins. Another study of catechin stereo-selectivity on membrane fluidity (Tsuchiya, 2001) shows that epicatechins (cis isomers) are more effective for reducing membrane fluidity than catechins (trans isomers). In the current study, it is interesting that in both cis and trans isomeric forms, the orientation of the hydroxyl or galloyl group at the 3-position of the C-ring is important and a wider binding energy difference can be observed, with the (−) isomers having a higher binding energy than the (+) isomers. Therefore, the current study proposed the (−) stereoisomer as a better inhibitor of wild-type EGFR than the corresponding (+) stereoisomer. The most studied catechin derivative is (−)-EGCG, which has increasingly been observed to play a crucial role as a therapeutic agent in various cancer conditions (Fujiki et al., 2018; Gan et al., 2018; Rady et al., 2018). In the current study, the gallated catechins and the (−) stereoisomer were proposed as better inhibitors of wild-type EGFR than their respective isomeric forms (−)-EGCG was the (−) stereoisomer of gallated catechin EGCG and had a high absolute value of binding energy. Thus, the current study also picked and proposed (−)-EGCG as a potential EGFR inhibitor and an anticancer agent. However (−)-EGCG showed a small decrease in binding affinity for the mutant L858R EGFR. Additionally, the MD simulation results of wild-type and mutant EGFR complexed with (−)-CG and (−)-EGCG indicated that the protein-ligand complexes were stable, and there were no significant changes in the protein conformation, especially the binding site, due to ligand binding. However, the results and conclusion drawn are from the computational study and experimental validations are warranted. Molecular docking faces several limitations that impact its accuracy. One significant constraint lies in the reliance on scoring functions, which may not consistently reflect the true binding free energy, leading to variations in results depending on the chosen scoring method. The assumption of rigid structures for ligands and receptors overlooks the inherent flexibility and dynamics of many biological molecules, potentially resulting in inaccuracies in predicting binding modes and affinities. Incomplete ligand conformational sampling poses a challenge as it may miss relevant conformations and hinder the identification of the correct binding pose. Ignoring solvent effects and using simplified solvation models can further contribute to inaccuracies, as the role of solvents in influencing binding interactions is often underestimated. Despite these limitations, molecular docking remains a valuable tool in drug discovery and structural biology. Integrating docking results with experimental data, thorough ligand conformational sampling, and employing multiple computational approaches can help mitigate some of these limitations and provide more reliable insights into molecular interactions. This study of the stereoisomers of catechins for the inhibition of kinase activity of the wild-type and the L858R mutant is one of its own kind and may aid in the general understanding of the stereochemical aspect of drugs, and help in designing novel stereoisomeric drugs with higher efficacy.

## Data Availability

The original contributions presented in the study are included in the article/Supplementary Material, further inquiries can be directed to the corresponding author.

## Supplementary material

SUPPLEMENTARY FIGURE S1 The stereoisomers of catechin derivatives docked to the EGFR kinase. The protein is shown as a ribbon representation in light orange. The ATP-binding site is shown on the surface and colored by element (O-atom, red; N-atom, blue; C-atom, white), whereas the stereoisomers of catechin derivatives and the bound reference ligand are shown as a stick representation colored by element (O-atom, red; N-atom, blue; C-atom, varying color). The balancing hydrogens of the compounds as stick representations are not shown for clarity.

SUPPLEMENTARY FIGURE S2 Binding poses of (−)-CG and (−)-EGCG with wild-type and mutant EGFR. The ligands and interacting residues are shown as a stick representation in yellow and gray, respectively. The yellow ligand (labeled with the ligand name) is in the center surrounded by the interacting residues (labeled with the residue name). The heteroatoms of ligands and interacting residues are shown in standard colors (e.g., O-atom, red; N-atom, blue). Hydrogen bonds are shown in cyan labeled with bond length (in Å).
