# Supplementary material for: Computational insights into the stereo-selectivity of catechins for the inhibition of the cancer therapeutic target EGFR kinase
Source: Front Pharmacol. 2024 Jan 11;14:1231671. doi: 10.3389/fphar.2023.1231671 (PMC10808699; doi:10.3389/fphar.2023.1231671)
Supplement: Supplementary file 1 [file DataSheet1.pdf]

Supplementary Material

Computational insights into the stereo-selectivity of catechins for the inhibition of the cancer therapeutic target EGFR kinase

Mohd Rehan*, Firoz Ahmed, Mohammad Imran Khan, Hifzur Rahman Ansari, Shazi Shakil, Moustafa E El-Araby, Salman Hosawi and Mohammad Saleem

*** Correspondence:** Corresponding Author: mrehan786@gmail.com, mrtahir@kau.edu.sa

Supplementary Figure S1. The stereoisomers of catechin derivatives docked to the EGFR kinase. The protein is shown as a ribbon representation in light orange. The ATP-binding site is shown on the surface and colored by element (O-atom, red; N-atom, blue; C-atom, hite), whereas the stereoisomers of catechin derivatives and the bound reference ligand are shown as a stick representation colored by element (O-atom, red; N-atom, blue; C-atom, varying color). The balancing hydrogens of the compounds as stick representations are not shown for clarity.

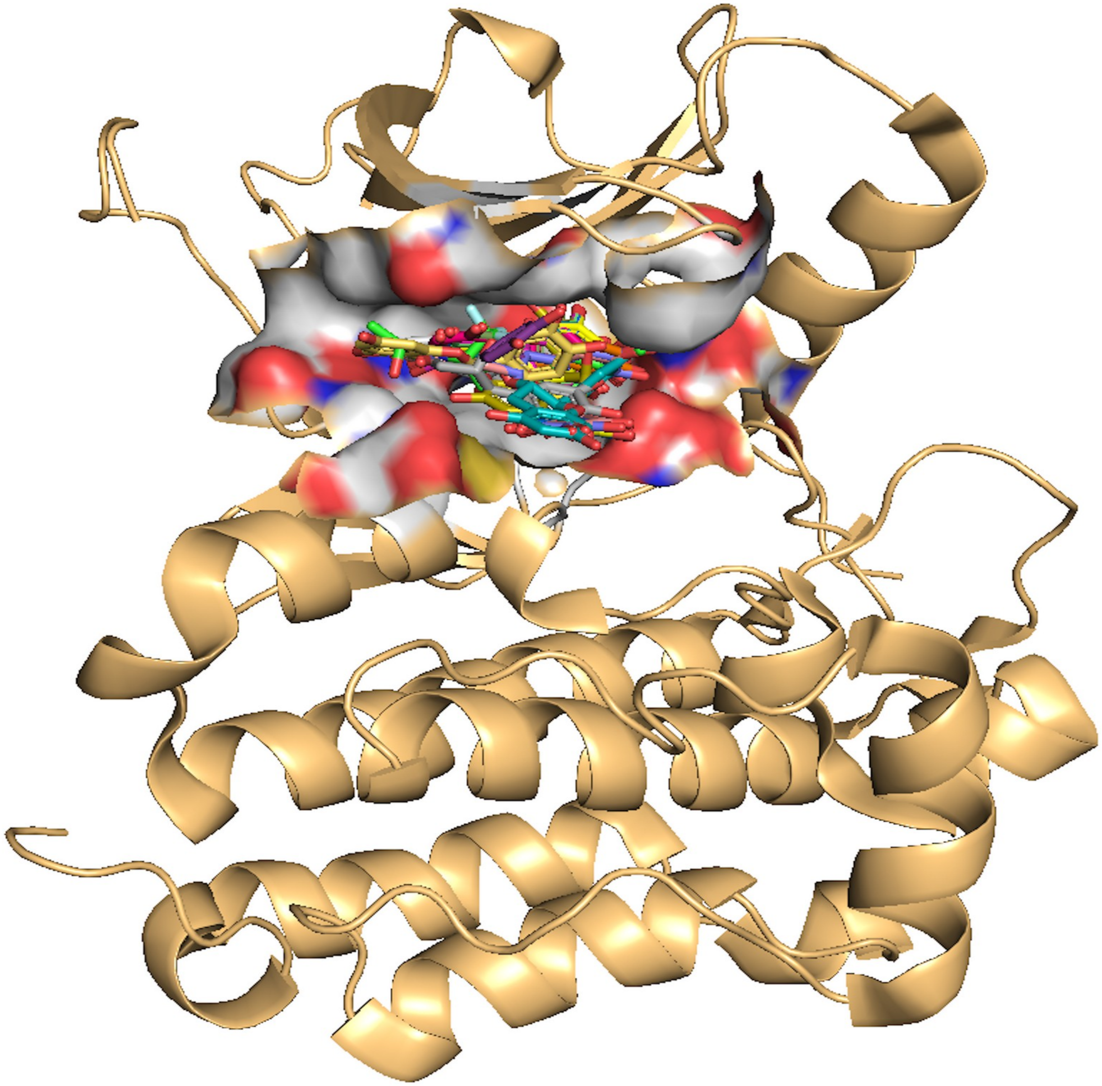

Supplementary Figure S2. Binding poses of (−)-CG and (−)-EGCG with wild-type and mutant EGFR. The ligands and interacting residues are shown as a stick representation in yellow and gray, respectively. The yellow ligand (labeled with the ligand name) is in the center surrounded by the interacting residues (labeled with the residue name). The heteroatoms of ligands and interacting residues are shown in standard colors (e.g., O-atom, red; N-atom, blue). Hydrogen bonds are shown in cyan labeled with bond length (in Å).

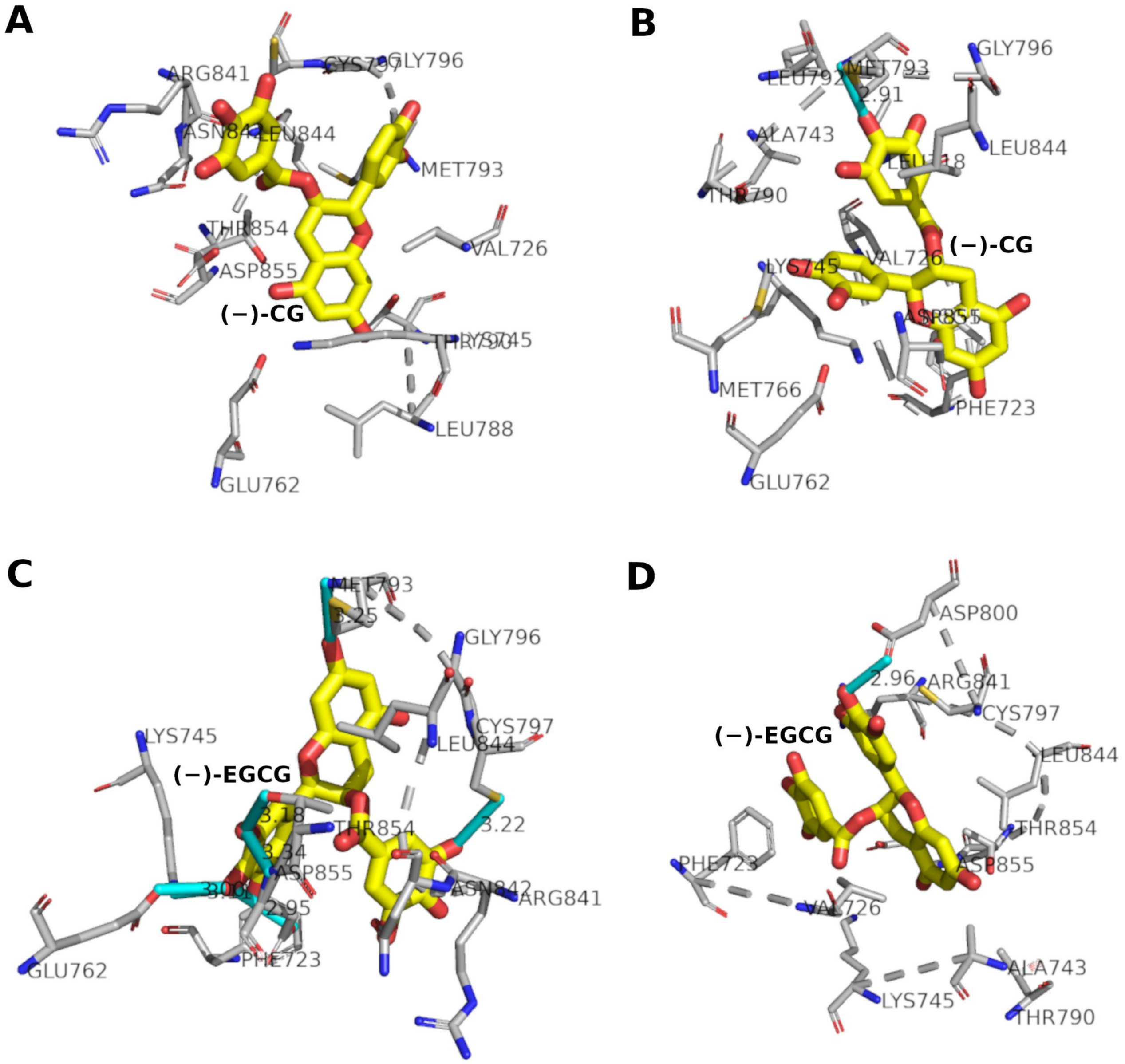

Supplementary Table S1. The stereoisomers of catechin derivatives as inhibitors of wild type EGFR kinase with the list of interacting residues. Each column represent interacting residues list for the compound name mentioned at the top. Each row represent a common interacting residue among the five selected compounds. The residues in bold are the interacting residues common with that of the native inhibitor.

**(−)-CG**	**(−)-GCG**	**(+)-CG**	**(−)-EGCG**	**(−)-ECG**	**(+)-GCG**	**(+)-ECG**	**(+)-EGCG**	**(−)-EGC**	**(−)-EC**	**(−)-C**	**(−)-GC**	**(+)-GC**	**(+)-C**	**(+)-EGC**	**(+)-EC**
**Leu-718**	**Leu-718**	-	-	-	**Leu-718**	-	**Leu-718**	**Leu-718**	**Leu-718**	-	**Leu-718**	**Leu-718**	-	**Leu-718**	-
-	-	-	-	-	-	-	-	-	Gly-719	-	-	Gly-719	-	-	-
-	-	-	**Phe-723**	**Phe-723**	**Phe-723**	**Phe-723**	**Phe-723**	-	-	-	-	-	**Phe-723**	-	-
Val-726	Val-726	Val-726	-	-	Val-726	-	Val-726	Val-726	Val-726	Val-726	-	Val-726	Val-726	-	Val-726
-	-	-	**Ala-743**	-	**Ala-743**	-	-	-	**Ala-743**	-	-	**Ala-743**	**Ala-743**	**Ala-743**	**Ala-743**
**Lys-745**	**Lys-745**	**Lys-745**	**Lys-745**	**Lys-745**	-	**Lys-745**	**Lys-745**	**Lys-745**	**Lys-745**	**Lys-745**	-	**Lys-745**	**Lys-745**	-	**Lys-745**
**Glu-762**	**Glu-762**	**Glu-762**	-	**Glu-762**	-	**Glu-762**	**Glu-762**	**Glu-762**	-	**Glu-762**	-	-	**Glu-762**	-	**Glu-762**
-	-	-	-	-	Met-766	-	Met-766	-	Met-766	-	Met-766	Met-766	-	-	-
-	-	-	-	-	**Cys-775**	-	-	-	-	-	**Cys-775**	-	-	-	-
Leu-788	Leu-788	Leu-788	-	-	-	-	Leu-788	Leu-788	Leu-788	Leu-788	-	Leu-788	-	-	Leu-788

Thr-790	Thr-790	Thr-790	-	-	Thr-790	-	Thr-790	Thr-790	Thr-790	Thr-790	Thr-790	Thr-790	Thr-790	-	Thr-790
-	-	-	-	-	Gln-791	-	-	-	-	-	-	-	-	-	-
-	-	-	Leu-792	-	-	-	-	-	-	-	-	-	Leu-792	Leu-792	-
Met-793	Met-793	Met-793	Met-793	Met-793	Met-793	Met-793	-	Met-793	-	-	Met-793	-	Met-793	Met-793	-
Gly-796	Gly-796	Gly-796	Gly-796	Gly-796	Gly-796	Gly-796	Gly-796	Gly-796	Gly-796	Gly-796	Gly-796	Gly-796	Gly-796	Gly-796	-
Cys-797	Cys-797	Cys-797	Cys-797	Cys-797	-	Cys-797	Cys-797	-	-	-	-	-	-	-	Cys-797
Arg-841	Arg-841	Arg-841	Arg-841	Arg-841	-	Arg-841	-	-	-	-	-	-	-	Arg-841	Arg-841
Asn-842	Asn-842	Asn-842	-	Asn-842	-	Asn-842	-	-	-	-	-	-	-	Asn-842	-
Leu-844	Leu-844	Leu-844	Leu-844	Leu-844	Leu-844	Leu-844	Leu-844	Leu-844	Leu-844	Leu-844	Leu-844	Leu-844	Leu-844	Leu-844	Leu-844
Thr-854	Thr-854	Thr-854	Thr-854	Thr-854	Thr-854	Thr-854	-	-	Thr-854	-	Thr-854	Thr-854	-	-	-
Asp-855	Asp-855	Asp-855	Asp-855	Asp-855	-	Asp-855	Asp-855	Asp-855	Asp-855	Asp-855	-	Asp-855	Asp-855	Asp-855	Asp-855

Supplementary Table S2. The stereoisomers of catechin derivatives as inhibitors of mutant L858R EGFR kinase with the list of interacting residues. Each column represent interacting residues list for the compound name mentioned at the top. Each row represent a common interacting residue among the five selected compounds. The residues in bold are the interacting residues common with that of the native inhibitor.

**(−)-CG**	**(−)-GCG**	**(+)-CG**	**(−)-EGCG**	**(−)-ECG**	**(+)-GCG**	**(+)-ECG**	**(+)-EGCG**	**(−)-EGC**	**(−)-EC**	**(−)-C**	**(−)-GC**	**(+)-GC**	**(+)-C**	**(+)-EGC**	**(+)-EC**
**Leu-718**	**Leu-718**	**Leu-718**	**-**	**Leu-718**	**Leu-718**	**-**	**-**	**-**	**-**	**-**	**-**	**Leu-718**	**Leu-718**	**-**	**-**
Phe-723	Phe-723	Phe-723	Phe-723	Phe-723	Phe-723	Phe-723	Phe-723	-	-	Phe-723	-	-	-	-	-
Val-726	Val-726	Val-726	Val-726	Val-726	Val-726	Val-726	Val-726	Val-726	Val-726	Val-726	Val-726	Val-726	Val-726	Val-726	Val-726
**Ala-743**	**Ala-743**	**Ala-743**	**Ala-743**	**Ala-743**	**Ala-743**	**-**	**Ala-743**	**Ala-743**	**-**	**Ala-743**	**Ala-743**	**Ala-743**	**Ala-743**	**Ala-743**	**Ala-743**
**Lys-745**	**Lys-745**	**Lys-745**	**Lys-745**	**Lys-745**	**Lys-745**	**Lys-745**	**Lys-745**	**Lys-745**	**Lys-745**	**Lys-745**	**Lys-745**	**Lys-745**	**Lys-745**	**Lys-745**	**Lys-745**
**Glu-762**	**Glu-762**	**Glu-762**	**-**	**Glu-762**	**Glu-762**	**-**	**-**	**Glu-762**	**Glu-762**	**Glu-762**	**Glu-762**	**-**	**-**	**Glu-762**	**Glu-762**
Met-766	Met-766	Met-766	-	Met-766	Met-766	Met-766	-	Met-766	Met-766	Met-766	Met-766	Met-766	Met-766	Met-766	Met-766
-	-	-	-	-	Leu-788	-	-	-	-	-	-	Leu-788	Leu-788	-	Leu-788
**Thr-790**	**Thr-790**	**Thr-790**	**Thr-790**	**Thr-790**	**Thr-790**	**Thr-790**	**Thr-790**	**Thr-790**	**Thr-790**	**Thr-790**	**Thr-790**	**Thr-790**	**Thr-790**	**Thr-790**	**Thr-790**
Leu-792	Leu-792	Leu-792	-	-	Leu-792	-	-	-	-	-	-	-	-	-	-

**Met-793**	**Met-793**	**Met-793**	-	-	**Met-793**	-	-	-	-	-	-	-	-	-	-
**Gly-796**	-	-	-	**Gly-796**	**Gly-796**	**Gly-796**	-	-	**Gly-796**	-	-	**Gly-796**	**Gly-796**	-	-
-	-	-	**Cys-797**	-	-	**Cys-797**	**Cys-797**	**Cys-797**	**Cys-797**	-	**Cys-797**	-	-	**Cys-797**	**Cys-797**
-	-	-	**Asp-800**	-	-	-	-	-	-	-	-	-	-	-	-
-	-	-	**Arg-841**	**Arg-841**	**Arg-841**	-	-	-	-	**Arg-841**	-	-	-	**Arg-841**	**Arg-841**
-	-	-	-	Asn-842	Asn-842	-	-	-	-	Asn-842	-	-	-	-	-
**Leu-844**	**Leu-844**	**Leu-844**	**Leu-844**	**Leu-844**	-	**Leu-844**	-	**Leu-844**	**Leu-844**	**Leu-844**	**Leu-844**	**Leu-844**	**Leu-844**	-	**Leu-844**
-	-	-	**Thr-854**	**Thr-854**	**Thr-854**	**Thr-854**	**Thr-854**	**Thr-854**	**Thr-854**	**Thr-854**	**Thr-854**	**Thr-854**	**Thr-854**	**Thr-854**	**Thr-854**
Asp-855	Asp-855	Asp-855	Asp-855	Asp-855	Asp-855	Asp-855	Asp-855	Asp-855	Asp-855	Asp-855	-	Asp-855	-	Asp-855	Asp-855

Supplementary Table S3. Hydrogen bond occupancy raw file (pairs.dat) for (−)-CG and (−)-EGCG with wild type and mutant EGFR complexes.

A. (−)-CG in complex with WILD type EGFR:

```
--> Results for readHBmap.py (Author: Ricardo O. S Soares)

--> Found 46 valid hydrogen bond pairs with occupancy of more than 0.0%

--> The hydrogen bond map (hbmap.xpm) has 10001 frames
```

Pair ID	donor-acceptor	Atom Number	Occupancy (%)	Pair ID
1	985NCG(H18) - 841ARG(O)	4715 - 2346	0.0	1
2	985NCG(H18) - 800ASP(OD2)	4715 - 1653	11.4	2
3	985NCG(H18) - 800ASP(OD1)	4715 - 1652	9.9	3
4	985NCG(H17) - 800ASP(OD2)	4714 - 1653	0.7	4
5	985NCG(H17) - 800ASP(OD1)	4714 - 1652	0.6	5
6	985NCG(H17) - 720SER(O)	4714 - 406	0.3	6
7	985NCG(H17) - 718LEU(O)	4714 - 388	8.4	7
8	985NCG(H16) - 855ASP(OD2)	4713 - 2576	1.3	8
9	985NCG(H16) - 855ASP(OD1)	4713 - 2575	0.1	9
10	985NCG(H16) - 842ASN(OD1)	4713 - 2355	25.1	10
11	985NCG(H16) - 841ARG(O)	4713 - 2346	6.4	11

12	985NCG(H16) - 800ASP(OD2)	4713 - 1653	24.1	12
13	985NCG(H16) - 800ASP(OD1)	4713 - 1652	21.4	13
14	985NCG(H15) - 793MET(O)	4712 - 1553	60.0	14
15	985NCG(H14) - 718LEU(O)	4711 - 388	0.3	15
16	985NCG(H13) - 793MET(O)	4710 - 1553	0.7	16
17	985NCG(H13) - 791GLN(O)	4710 - 1517	0.0	17
18	985NCG(H10) - 790THR(N)	4707 - 1487	0.0	18
19	985NCG(H10) - 788LEU(O)	4707 - 1467	1.4	19
20	985NCG(H10) - 762GLU(OE2)	4707 - 1071	45.3	20
21	985NCG(H10) - 762GLU(OE1)	4707 - 1070	16.3	21
22	985NCG(H10) - 743ALA(O)	4707 - 762	2.5	22
23	985NCG(H9) - 855ASP(OD2)	4706 - 2576	0.2	23
24	985NCG(H9) - 855ASP(OD1)	4706 - 2575	0.9	24
25	985NCG(H9) - 762GLU(OE2)	4706 - 1071	19.5	25
26	985NCG(H9) - 762GLU(OE1)	4706 - 1070	13.0	26
27	856PHE(HN) - 985NCG(O3)	2580 - 4667	0.0	27
28	855ASP(HN) - 985NCG(O3)	2568 - 4667	0.0	28
29	854THR(HG1) - 985NCG(O7)	2560 - 4671	12.3	29
30	854THR(HG1) - 985NCG(O4)	2560 - 4668	0.1	30

31	854THR(HG1) - 985NCG(O3)	2560 - 4667	0.3	31
32	842ASN(D21) - 985NCG(O7)	2357 - 4671	2.7	32
33	842ASN(D21) - 985NCG(O3)	2357 - 4667	0.3	33
34	841ARG(H11) - 985NCG(O9)	2340 - 4673	5.5	34
35	841ARG(HE) - 985NCG(O9)	2337 - 4673	0.0	35
36	797CYS(HN) - 985NCG(O9)	1596 - 4673	0.6	36
37	797CYS(HN) - 985NCG(O7)	1596 - 4671	0.2	37
38	796GLY(HN) - 985NCG(O8)	1589 - 4672	0.0	38
39	793MET(HN) - 985NCG(O8)	1538 - 4672	0.5	39
40	793MET(HN) - 985NCG(O5)	1538 - 4669	89.4	40
41	790THR(HG1) - 985NCG(O4)	1494 - 4668	0.5	41
42	745LYS(HZ1) - 985NCG(O4)	799 - 4668	0.0	42
43	745LYS(HZ1) - 985NCG(O3)	799 - 4667	1.0	43
44	745LYS(HN) - 985NCG(O4)	783 - 4668	0.0	44
45	720SER(HN) - 985NCG(O10)	397 - 4674	0.0	45
46	719GLY(HN) - 985NCG(O6)	390 - 4670	0.0	46

==

B.(−)-CG in complex with mutant EGFR:

--> Results for readHBmap.py (Author: Ricardo O. S Soares)

--> Found 37 valid hydrogen bond pairs with occupancy of more than 0.0%

--> The hydrogen bond map (hbmap.xpm) has 10001 frames

Pair ID	donor-acceptor	Atom Number	Occupancy (%)	Pair ID
1	985NCG(H18) - 793MET(O)	4563 - 1504	31.4	1
2	985NCG(H18) - 791GLN(O)	4563 - 1468	0.1	2
3	985NCG(H17) - 793MET(O)	4562 - 1504	0.0	3
4	985NCG(H17) - 718LEU(O)	4562 - 388	0.0	4
5	985NCG(H16) - 793MET(O)	4561 - 1504	0.2	5
6	985NCG(H16) - 791GLN(O)	4561 - 1468	18.3	6
7	985NCG(H15) - 788LEU(O)	4560 - 1418	3.1	7
8	985NCG(H15) - 743ALA(O)	4560 - 762	3.2	8
9	985NCG(H14) - 855ASP(OD2)	4559 - 2527	21.2	9
10	985NCG(H14) - 855ASP(OD1)	4559 - 2526	12.1	10
11	985NCG(H14) - 762GLU(OE1)	4559 - 1021	0.0	11
12	985NCG(H11) - 855ASP(OD2)	4556 - 2527	21.0	12

13	985NCG(H11) - 855ASP(OD1)	4556 - 2526	5.4	13
14	985NCG(H11) - 842ASN(OD1)	4556 - 2306	0.1	14
15	985NCG(H11) - 723PHE(O)	4556 - 443	0.0	15
16	985NCG(H10) - 842ASN(OD1)	4555 - 2306	0.6	16
17	985NCG(H10) - 841ARG(O)	4555 - 2297	28.7	17
18	985NCG(H10) - 800ASP(OD2)	4555 - 1604	0.0	18
19	985NCG(H10) - 800ASP(OD1)	4555 - 1603	0.0	19
20	855ASP(HN) - 985NCG(O5)	2519 - 4518	0.2	20
21	854THR(HG1) - 985NCG(O5)	2511 - 4518	20.6	21
22	842ASN(D21) - 985NCG(O4)	2308 - 4517	0.3	22
23	842ASN(D21) - 985NCG(O3)	2308 - 4516	0.1	23
24	842ASN(D21) - 985NCG(O1)	2308 - 4514	0.1	24
25	841ARG(H21) - 985NCG(O4)	2294 - 4517	0.5	25
26	841ARG(H11) - 985NCG(O4)	2291 - 4517	0.4	26
27	841ARG(HE) - 985NCG(O4)	2288 - 4517	0.3	27
28	796GLY(HN) - 985NCG(O10)	1540 - 4523	0.0	28
29	793MET(HN) - 985NCG(O10)	1489 - 4523	57.2	29
30	793MET(HN) - 985NCG(O8)	1489 - 4521	35.7	30
31	790THR(HG1) - 985NCG(O8)	1445 - 4521	0.0	31

32	790THR(HG1) - 985NCG(O7)	1445 - 4520	0.0	32
33	745LYS(HZ1) - 985NCG(O5)	799 - 4518	0.3	33
34	745LYS(HZ1) - 985NCG(O1)	799 - 4514	0.2	34
35	745LYS(HN) - 985NCG(O7)	783 - 4520	0.2	35
36	723PHE(HN) - 985NCG(O4)	425 - 4517	0.0	36
37	722ALA(HN) - 985NCG(O4)	415 - 4517	0.3	37

===

C.(-)-EGCG in complex with WILD type EGFR:

--> Results for readHBmap.py (Author: Ricardo O. S Soares)

--> Found 55 valid hydrogen bond pairs with occupancy of more than 0.0%

--> The hydrogen bond map (hbmap.xpm) has 10001 frames

Pair ID	donor-acceptor	Atom Number	Occupancy (%)	Pair ID
1	985egc(H18) - 855ASP(OD2)	4715 - 2576	7.4	1
2	985egc(H18) - 855ASP(OD1)	4715 - 2575	9.7	2
3	985egc(H18) - 842ASN(OD1)	4715 - 2355	15.1	3
4	985egc(H18) - 841ARG(O)	4715 - 2346	6.8	4

5	985egc(H18) - 720SER(O)	4715 -	406	0.2	5
6	985egc(H17) - 855ASP(OD2)	4714 -	2576	6.7	6
7	985egc(H17) - 855ASP(OD1)	4714 -	2575	9.1	7
8	985egc(H17) - 842ASN(OD1)	4714 -	2355	0.0	8
9	985egc(H17) - 841ARG(O)	4714 -	2346	0.4	9
10	985egc(H17) - 800ASP(OD1)	4714 -	1652	0.0	10
11	985egc(H16) - 855ASP(OD2)	4713 -	2576	22.6	11
12	985egc(H16) - 855ASP(OD1)	4713 -	2575	30.6	12
13	985egc(H16) - 842ASN(O)	4713 -	2360	0.1	13
14	985egc(H16) - 842ASN(OD1)	4713 -	2355	7.3	14
15	985egc(H16) - 841ARG(O)	4713 -	2346	28.6	15
16	985egc(H15) - 855ASP(OD2)	4712 -	2576	0.1	16
17	985egc(H15) - 855ASP(OD1)	4712 -	2575	3.3	17
18	985egc(H15) - 762GLU(OE2)	4712 -	1071	53.3	18
19	985egc(H15) - 762GLU(OE1)	4712 -	1070	35.3	19
20	985egc(H14) - 855ASP(OD2)	4711 -	2576	2.5	20
21	985egc(H14) - 855ASP(OD1)	4711 -	2575	3.6	21
22	985egc(H14) - 762GLU(OE2)	4711 -	1071	49.9	22
23	985egc(H14) - 762GLU(OE1)	4711 -	1070	31.6	23

24	985egc(H13) - 788LEU(O)	4710 -	1467	1.1	24
25	985egc(H13) - 762GLU(OE1)	4710 -	1070	0.2	25
26	985egc(H13) - 743ALA(O)	4710 -	762	0.0	26
27	985egc(H10) - 793MET(O)	4707 -	1553	0.3	27
28	985egc(H10) - 791GLN(O)	4707 -	1517	0.0	28
29	985egc(H9) - 718LEU(O)	4706 -	388	0.1	29
30	856PHE(HN) - 985egc(O6)	2580 -	4670	0.0	30
31	855ASP(HN) - 985egc(O6)	2568 -	4670	0.5	31
32	854THR(HG1) - 985egc(O8)	2560 -	4672	0.0	32
33	854THR(HG1) - 985egc(O6)	2560 -	4670	2.7	33
34	854THR(HG1) - 985egc(O5)	2560 -	4669	0.7	34
35	842ASN(D21) - 985egc(O11)	2357 -	4675	0.1	35
36	842ASN(D21) - 985egc(O10)	2357 -	4674	0.0	36
37	842ASN(D21) - 985egc(O9)	2357 -	4673	0.5	37
38	842ASN(D21) - 985egc(O7)	2357 -	4671	4.1	38
39	841ARG(H21) - 985egc(O11)	2343 -	4675	0.0	39
40	841ARG(H21) - 985egc(O9)	2343 -	4673	0.0	40
41	841ARG(H11) - 985egc(O11)	2340 -	4675	1.5	41
42	841ARG(H11) - 985egc(O10)	2340 -	4674	0.2	42

43	841ARG(H11) - 985egc(O9)	2340 - 4673	0.1	43
44	841ARG(HE) - 985egc(O11)	2337 - 4675	0.2	44
45	841ARG(HE) - 985egc(O10)	2337 - 4674	0.0	45
46	797CYS(HN) - 985egc(O3)	1596 - 4667	0.0	46
47	793MET(HN) - 985egc(O4)	1538 - 4668	61.3	47
48	790THR(HG1) - 985egc(O5)	1494 - 4669	51.4	48
49	790THR(HN) - 985egc(O5)	1488 - 4669	0.0	49
50	745LYS(HZ1) - 985egc(O9)	799 - 4673	0.1	50
51	745LYS(HZ1) - 985egc(O8)	799 - 4672	0.0	51
52	745LYS(HZ1) - 985egc(O7)	799 - 4671	0.0	52
53	745LYS(HZ1) - 985egc(O6)	799 - 4670	2.4	53
54	745LYS(HN) - 985egc(O5)	783 - 4669	1.7	54
55	720SER(HN) - 985egc(O10)	397 - 4674	0.0	55

==

D.(−)−EGCG in complex with mutant EGFR:

--> Results for readHBmap.py (Author: Ricardo O. S Soares)

--> Found 70 valid hydrogen bond pairs with occupancy of more than 0.0%

--> The hydrogen bond map (hbmap.xpm) has 10001 frames

Pair ID	donor-acceptor	Atom Number	Occupancy (%)	Pair ID
1	985EGC(H17) - 855ASP(OD2)	4563 - 2527	0.0	1
2	985EGC(H17) - 855ASP(OD1)	4563 - 2526	0.2	2
3	985EGC(H17) - 721GLY(O)	4563 - 413	0.1	3
4	985EGC(H17) - 720SER(O)	4563 - 406	16.4	4
5	985EGC(H16) - 841ARG(NH1)	4562 - 2290	0.0	5
6	985EGC(H16) - 837ASP(OD2)	4562 - 2232	10.1	6
7	985EGC(H16) - 837ASP(OD1)	4562 - 2231	1.2	7
8	985EGC(H16) - 721GLY(O)	4562 - 413	0.0	8
9	985EGC(H16) - 720SER(O)	4562 - 406	7.0	9
10	985EGC(H15) - 800ASP(OD2)	4561 - 1604	37.4	10
11	985EGC(H15) - 800ASP(OD1)	4561 - 1603	49.7	11
12	985EGC(H14) - 800ASP(OD2)	4560 - 1604	37.5	12
13	985EGC(H14) - 800ASP(OD1)	4560 - 1603	47.3	13
14	985EGC(H14) - 718LEU(O)	4560 - 388	0.1	14
15	985EGC(H13) - 800ASP(OD2)	4559 - 1604	0.2	15
16	985EGC(H13) - 800ASP(OD1)	4559 - 1603	3.0	16

17	985EGC(H10) - 791GLN(O)	4556 - 1468	1.0	17
18	985EGC(H10) - 724GLY(O)	4556 - 450	43.3	18
19	985EGC(H10) - 723PHE(O)	4556 - 443	0.4	19
20	985EGC(H10) - 720SER(O)	4556 - 406	0.0	20
21	985EGC(H10) - 719GLY(O)	4556 - 395	4.4	21
22	985EGC(H10) - 718LEU(O)	4556 - 388	44.2	22
23	985EGC(H9) - 855ASP(OD2)	4555 - 2527	0.3	23
24	985EGC(H9) - 855ASP(OD1)	4555 - 2526	0.5	24
25	985EGC(H9) - 842ASN(OD1)	4555 - 2306	0.0	25
26	985EGC(H9) - 724GLY(O)	4555 - 450	5.1	26
27	985EGC(H9) - 723PHE(O)	4555 - 443	16.3	27
28	985EGC(H9) - 721GLY(O)	4555 - 413	0.0	28
29	858ARG(H21) - 985EGC(O9)	2577 - 4522	2.7	29
30	858ARG(H11) - 985EGC(O9)	2574 - 4522	1.3	30
31	842ASN(D21) - 985EGC(O10)	2308 - 4523	0.0	31
32	842ASN(D21) - 985EGC(O7)	2308 - 4520	12.7	32
33	841ARG(H21) - 985EGC(O11)	2294 - 4524	0.0	33
34	841ARG(H21) - 985EGC(O10)	2294 - 4523	0.1	34
35	841ARG(H21) - 985EGC(O9)	2294 - 4522	0.0	35

36	841ARG(H21) - 985EGC(O7)	2294 - 4520	13.6	36
37	841ARG(H21) - 985EGC(O5)	2294 - 4518	0.1	37
38	841ARG(H11) - 985EGC(O7)	2291 - 4520	0.0	38
39	841ARG(H11) - 985EGC(O5)	2291 - 4518	0.1	39
40	841ARG(H11) - 985EGC(O3)	2291 - 4516	0.0	40
41	841ARG(HE) - 985EGC(O11)	2288 - 4524	0.0	41
42	841ARG(HE) - 985EGC(O7)	2288 - 4520	0.1	42
43	841ARG(HE) - 985EGC(O5)	2288 - 4518	1.9	43
44	803ARG(H21) - 985EGC(O8)	1664 - 4521	0.2	44
45	803ARG(H21) - 985EGC(O6)	1664 - 4519	0.1	45
46	803ARG(H11) - 985EGC(O8)	1661 - 4521	0.2	46
47	803ARG(H11) - 985EGC(O6)	1661 - 4519	0.1	47
48	800ASP(HN) - 985EGC(O6)	1596 - 4519	0.0	48
49	797CYS(HN) - 985EGC(O8)	1547 - 4521	0.2	49
50	797CYS(HN) - 985EGC(O6)	1547 - 4519	0.0	50
51	797CYS(HN) - 985EGC(O5)	1547 - 4518	0.3	51
52	745LYS(HZ1) - 985EGC(O3)	799 - 4516	0.2	52
53	726VAL(HN) - 985EGC(O4)	466 - 4517	38.6	53
54	724GLY(HN) - 985EGC(O4)	445 - 4517	0.0	54

55	724GLY(HN) – 985EGC(O3)	445 – 4516	0.0	55
56	723PHE(HN) – 985EGC(O10)	425 – 4523	0.0	56
57	723PHE(HN) – 985EGC(O4)	425 – 4517	0.3	57
58	723PHE(HN) – 985EGC(O3)	425 – 4516	1.0	58
59	722ALA(HN) – 985EGC(O10)	415 – 4523	0.3	59
60	722ALA(HN) – 985EGC(O9)	415 – 4522	0.1	60
61	722ALA(HN) – 985EGC(O7)	415 – 4520	0.0	61
62	722ALA(HN) – 985EGC(O3)	415 – 4516	0.3	62
63	721GLY(HN) – 985EGC(O4)	408 – 4517	3.9	63
64	720SER(HG1) – 985EGC(O10)	404 – 4523	0.1	64
65	720SER(HG1) – 985EGC(O9)	404 – 4522	0.0	65
66	720SER(HN) – 985EGC(O9)	397 – 4522	0.2	66
67	720SER(HN) – 985EGC(O6)	397 – 4519	0.0	67
68	720SER(HN) – 985EGC(O5)	397 – 4518	0.1	68
69	720SER(HN) – 985EGC(O4)	397 – 4517	0.0	69
70	720SER(HN) – 985EGC(O1)	397 – 4514	0.2	70
